# Density Peak clustering of protein sequences associated to a Pfam clan reveals clear similarities and interesting differences with respect to manual family annotation

**DOI:** 10.1186/s12859-021-04013-x

**Published:** 2021-03-12

**Authors:** Elena Tea Russo, Alessandro Laio, Marco Punta

**Affiliations:** 1 SISSA, 34136 Trieste, Italy; 2 Centre for Evolution and Cancer, The Institute of Cancer Research, London, SM2 5NG UK; 3 Present Address: Center for Omics Sciences, IRCCS San Raffaele Hospital, 20132 Milan, Italy

**Keywords:** Protein families, Pfam, Unsupervised clustering, Sequence analysis

## Abstract

**Background:**

The identification of protein families is of outstanding practical importance for in silico protein annotation and is at the basis of several bioinformatic resources. Pfam is possibly the most well known protein family database, built in many years of work by domain experts with extensive use of manual curation. This approach is generally very accurate, but it is quite time consuming and it may suffer from a bias generated from the hand-curation itself, which is often guided by the available experimental evidence.

**Results:**

We introduce a procedure that aims to identify automatically putative protein families. The procedure is based on Density Peak Clustering and uses as input only local pairwise alignments between protein sequences. In the experiment we present here, we ran the algorithm on about 4000 full-length proteins with at least one domain classified by Pfam as belonging to the Pseudouridine synthase and Archaeosine transglycosylase (PUA) clan. We obtained 71 automatically-generated sequence clusters with at least 100 members. While our clusters were largely consistent with the Pfam classification, showing good overlap with either single or multi-domain Pfam family architectures, we also observed some inconsistencies. The latter were inspected using structural and sequence based evidence, which suggested that the automatic classification captured evolutionary signals reflecting non-trivial features of protein family architectures. Based on this analysis we identified a putative novel pre-PUA domain as well as alternative boundaries for a few PUA or PUA-associated families. As a first indication that our approach was unlikely to be clan-specific, we performed the same analysis on the P53 clan, obtaining comparable results.

**Conclusions:**

The clustering procedure described in this work takes advantage of the information contained in a large set of pairwise alignments and successfully identifies a set of putative families and family architectures in an unsupervised manner. Comparison with the Pfam classification highlights significant overlap and points to interesting differences, suggesting that our new algorithm could have potential in applications related to automatic protein classification. Testing this hypothesis, however, will require further experiments on large and diverse sequence datasets.

**Supplementary Information:**

The online version contains supplementary material available at 10.1186/s12859-021-04013-x.

## Background

Conserved evolutionary modules shared by different proteins typically present some degree of structural and, to a lesser extent, functional similarity [1]. These modules are commonly called domains, and the ensemble of sequences from a set of evolutionarily related domains is called a family. Identification of domains and families is of great importance in bioinformatics: if a protein of known sequence but unknown function and/or structure is annotated by a tool capable of recognizing its relationship to an annotated family, or to several families organized in an architecture, one can hope to approximately infer from this information a possible biochemical or cellular role for the protein [2]. Several resources have been developed toward this goal, including but not limited to, Pfam [3], SMART [4], TIGRFAMs [5], PANTHER [6], SFLD [7], CATH-Gene3D [8], SUPERFAMILY [9] and ECOD [10]. Some databases restrict themselves to specific functional categories (SMART, SFLD), phylogenetic groups (TIGRFAMs) or to families for which structural information is available (CATH-Gene3D, SUPERFAMILY, ECOD). Others aim to classify the protein sequence space more widely (Pfam, PANTHER). Most databases try to identify domains (evolutionary, structural and/or functional units) while some build families for full-length protein sequences (TIGRFAM, PANTHER). All of these resources take advantage, at some level, of expert manual curation. While this helps increasing the quality of families, it limits the proportion of the sequence space that can be covered by a classification scheme. For example, Pfam residue coverage of the UniProtKB database as of release 04/2018 [3] was around 53% with more than 20% of all UniProtKB sequences lacking any type of Pfam annotation. In order to alleviate this problem, databases have been developed integrating classifications from several resources into a single platform (InterPro [11], CDD [12]).

An alternative approach to manually curated family classification is performing automatic, sequence-based classification of protein regions. Automated family classification has a long history in protein bioinformatics and over the years has led to the development of algorithms such as ADDA [13], COG [14], EVEREST [15], CD-HIT [16], linclust [17], UCLUST [18] and MCL [19], among others. Most of these methods aim to find conserved family architectures (i.e., full-length sequence homologs). To our knowledge ADDA and EVEREST are the only ones that were specifically developed to identify individual families. EVEREST uses Pfam information to infer the general notion of ”protein family” via a supervised learning step [15] while the ADDA clustering algorithm uses elaborate models to extract information from the sequence space and define domain boundaries [13]. The published implementations of these two algorithms have not been maintained in the last years and are thus obsolete with respect to current operating systems. Until 2015, ADDA was used to produce Pfam-B: this is an automatically-built companion to the manually curated Pfam main family collection, which identifies novel entries not documented in Pfam-A. Between 2015 and 2020, Pfam-B was discontinued. Only recently, Pfam-B has been resurrected (see Pfam blog: https://xfam.wordpress.com/2020/06/30/a-new-pfam-b-is-released/ ). However, the fine details of the clustering procedure that has been adopted are, at the moment of writing, not available.

Finally, algorithms based on more sophisticated concepts such as k-mers and deep learning have been recently developed to project full protein sequences into interpretable representations. As an example, [20] uses k-mers to annotate the PRX protein superfamily; [21] instead uses recurrent neural networks to learn from UniRef50 a vector representation of proteins, capturing a subset of known protein characteristics.

Our hypothesis is that, with the current size of protein sequence databases, it is possible in many cases to use information derived exclusively from sequence alignments to automatically identify protein families. Our approach is based on Density Peak Clustering (DPC) [22], an algorithm which clusters together data based on their local density in a non-parametric manner. This clustering approach is appropriate for protein sequence analysis since it requires estimating only the distance between the data points with no use of their coordinates.

In the problem of protein sequence classification the alignment score obtained by pairwise sequence alignment is a natural choice for defining ’closeness’ between sequences. However, in the task of family identification one cannot perform clustering using simply the alignment score. Proteins can often contain several families, whose ordered succession defines an architecture, e.g. -A-B- or -A-C-D-. Any approach aimed at finding families must be able to take into account the fact that two sequences can be extremely similar in a region, but totally different in the rest of the sequence: for example, two proteins of architecture –A–B– and –A–C–D– are similar only in region A. For the same reason, a sequence can be separately similar to two other sequences which are not similar at all between each other. To resolve these difficulties, we focus on clustering *local* pairwise alignments, and use distances between alignments defined considering their boundaries, rather than their score. The distance we use captures the difference between an alignment covering only region A, one covering only region B and a third covering both regions A-B. If there are enough alignments in which A and B are not covered together, a clustering algorithm such as DPC applied to those distances should be able, at least in principle, to recognize family A and family B as different objects.

We call the set of sequences where we want to identify the families the “query set”. The first step of the algorithm requires running BLAST [23] alignments of all the query set against a large database of sequences, which we call the “search set” and is roughly represented by a redundancy-reduced version of UniProtKB. Next, independently for each query sequence, we identify all regions that align to sequences in the search set. Query regions found in the alignments can be significantly smaller than the full sequence, and are typically many thousands, strongly overlapping with each other. We group them together by DPC, obtaining what we call “primary clusters”, which provide a first approximation of the architecture of the query sequence, with each cluster potentially corresponding to a separate domain. Primary clusters of different query sequences are then grouped into ‘metaclusters” (MCs) based on the number of search sequence regions they have in common. This step is performed once again with DPC and further MC merging. This corresponds to grouping together the individual query domain-like regions identified in the previous step into families. The sequence regions that form a metacluster are regions belonging to several hundred different proteins or more (we consider MCs of size > 100) which typically have a relatively high similarity between each other. This sets of sequences can be used as seeds to build a Multiple Sequence Alignment (MSA) and a corresponding profile-HMM [24], similarly to what is done in Pfam [3].

In the experiment we perform here, we use the procedure outlined above to analyze a set of about 4000 full-length sequences and perform manual validation of the results by comparison to the Pfam annotation and to available structures. The dataset we consider, in particular, is constituted of sequences that contain at least one family from the Pfam defined Pseudouridine synthase and Archaeosine transglycosylase (PUA) clan [25]. In a second experiment, discussed in the final paragraph of the “Results” section, we run our procedure on sequences from the P53 Pfam clan [26]. In the Pfam classification, clans (also known as “superfamilies” in other databases) group together families that are evolutionary related. Families in Pfam clans may be remotely related (possibly representing domains of different function) or, sometimes, evolutionarily close (i.e., sharing a sizable number of member regions). As of Pfam v.31, which we use throughout unless otherwise specified, the PUA clan comprised the following 11 families (25,659 sequences in total): ASCH, DUF3850, EVE, LON_substr_bdg, Methyltranf_PUA, PUA, PUA_2, TruB-C_2, TruB_C, UPF0113 and YTH. We choose the PUA clan for several reasons. PUA is a medium size clan, thus rendering in-depth manual analysis of results more manageable while still providing a rather complex set of relationships between sequences within and outside of the clan; moreover, extensive structural information is available for most of these families, which provides crucial insight for evaluating *a posteriori* the quality of a classification. Additionally, the PUA clan is well-known to us from previous studies [27]. We name the dataset of query proteins PUA_UR50 (more details on how it is generated are given in the “Methods” section). This dataset contains proteins with a large variety of architectures, including also numerous families which are not in the PUA clan. We will show that our procedure allows identifying both PUA and non-PUA families within the dataset.

## Results

We first describe the measures we developed to compare Metaclusters (MCs) to Pfam families and clans; then we proceed to present in detail the results obtained.

**Fig. 1:**
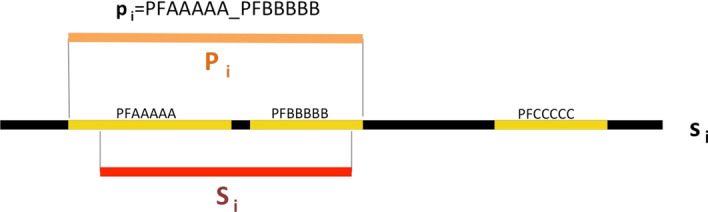
Schematic representation of Pfam Ground Truth Architecture (GTA) assignment to a generic protein region ${{\mathcal {S}}}_i$. In this example, the full-length protein $s_i$ has the following three-family architecture: PFAAAAA + PFBBBBB + PFCCCCC; the aligned region of the search sequence, ${{\mathcal {S}}}_i$, covers (partially) only PFAAAAA and PFBBBBB; thus, the Pfam ground truth of $S_i$ is $p_i$ = PFAAAAA_PFBBBBB (note that a 1-residue overlap of ${\mathcal {S}}_i$ with a family is enough for the latter to be included into the GTA); in orange we show $\mathcal {P}_i$, namely the full region covered by the GTA families on sequence $s_i$, including residues between them

### In-house Pfam annotation of the UniRef50 database and definition of dominant ground truth architecture of a metacluster

As previously mentioned, our protein database of reference is UniRef50 (v. 2017/7) published by the UniProt consortium and obtained by reducing at 50% sequence identity (using CD-HIT [16]) the redundancy in the UniRefKB database. Since not all sequences in UniRef50 are annotated in Pfam, we are not able to use the Pfam database family assignments directly. Instead, we run each sequence in UniRef50 against the set of all Pfam_A.hmm models (v. 31) using the hmmscan program from the HMMER 3.1b2 suite [24]. We assign to each protein sequence a Pfam family architecture according to the models’ manually-curated gathering thresholds. In the case of multiple significant matches overlapping along the same protein sequence, we keep only the Pfam annotation corresponding to the lowest E-value. Overlaps are calculated using start and end alignment positions. Note that this protocol does not account for domain nesting. We define the Pfam Ground Truth Architecture (GTA) $p_i$ of a region ${\mathcal {S}}_i$ as the ordered set of Pfam families that has overlap of at least one amino acid with ${\mathcal {S}}_i$, if any. The order of the families reflects their relative position along ${\mathcal {S}}_i$. For example, suppose that we want to determine the GTA of the region ${\mathcal {S}}_i$ of protein $s_i=$Q5BH58 spanning positions 132 to 567. Pfam annotation for Q5BH58 is as follows: PF02190 (aa 10-258), PF00004 (aa 482-625), PF05362 (aa 706-915). In this case, the GTA of ${\mathcal {S}}_i$ is represented by $p_i=$PF02190_PF00004. We can alternatively define the GTA in terms of Pfam clans to which each Pfam family is associated (in this case $p_i(clan)=$CL0178_CL0023); again, the GTA is an ordered string of (clan) ids. If a family is not associated to a clan in Pfam, we use the family id also in the clan’s GTA. $\mathcal {P}_i$ is the whole region of protein $s_i$ covered by the Pfam families of the GTA, including every residue between them (see Fig. 1). In the example above the $\mathcal {P}_i$ of ${\mathcal {S}}_i$ is the interval between residue 10 and 625.

Next, we define the Pfam Dominant ground truth Architecture (we will abbreviate it as DA) of a metacluster as the most abundant GTA among all the sequence regions belonging to a metacluster. The DA can be defined at the family (using the $p_i$s) or clan level (using the $p_i(clan)$s).

### Comparing metaclusters with the Pfam “ground truth”

When comparing Pfam annotations to our MC classification, one should take into account the following: (1) evolutionary distances between families within a Pfam clan can differ greatly; in particular, some families may be very closely related to each other. For this reason, it is often more informative to look at consistency of annotation in MCs at the clan level; (2) along with many full-length sequences, UniRef50 also contains sequence fragments. This may be relevant when comparing MC member annotations, especially for those MCs with a multi-domain DA. (3) Pfam classification of families and clans can be incomplete; as a consequence, regions in UniRef50 that are not currently annotated in Pfam may still belong to known Pfam families and clans.

Given a MC, we first determine its DA both at the family and at the clan level and we indicate with %DAF (family) and %DAC (clan) their relative frequencies among MC members. Hereafter, we call “DA members” those member regions for which, at the clan level, the GTA coincides with the DA. Next, we consider MC members that match the DA (again, at the clan level) only partially. While this makes sense in light of observations (2) and (3) above, it also allows for some variability in length among MC members. We compute the percentage of MC members with a GTA that lacks one or more of the DA clans but, at the same time, doesn’t feature any extra clan(s). We sum this percentage to %DAC and report it as %DACF (F = fewer); we still ask that the remaining clans are in the same order as in the DA. Note that MC members lacking any Pfam annotation are counted in %DACF. This is consistent with the idea that having no Pfam annotation does not imply that a region is not part of an existing Pfam clan [observation (3) above]. Finally, we compute the percentage of MC members with a GTA that features one or more Pfam clans not found in the DA but, at the same time, contains at least one of the original DA clans. We sum this to %DACF and call it %DACFA (A = additional). We will see that the analysis of differences between these percentage scores facilitates the identification of MCs that may not be evolutionarily sound as well as those MCs that may help improving the Pfam classification by expanding family and clan membership, by uncovering novel domains or by pointing to potential inconsistencies in the existing annotation. Comparison between the DPC and Pfam classifications cannot be reduced to presence or absence of families and clans on MC members. Indeed, the degree of agreement between the boundaries of ${\mathcal {S}}_i$ of the MCs’ regions and the boundaries of $\mathcal {P}_i$ of the Pfam annotations is also important. For the sake of the comparison between MC and Pfam family boundaries, we define:

1 $$\begin{aligned} F_{red,i}= & {} \frac{ |\mathcal {P}_i \setminus [{\mathcal {S}}_i \cap \mathcal {P}_i] | }{ |\mathcal {P}_i| } \end{aligned}$$

2 $$\begin{aligned} F_{ext,i}= & {} \frac{ |{\mathcal {S}}_i \setminus [{\mathcal {S}}_i \cap \mathcal {P}_i] | }{ |{\mathcal {S}}_i| } \end{aligned}$$

$F_{red,i}$ represents the fraction of the DA $\mathcal {P}_i$ that is not covered by the region ${\mathcal {S}}_i$; vice versa, $F_{ext,i}$ is the fraction of the region ${\mathcal {S}}_i$ that is not covered by the DA. We use these two measures to characterize boundaries of entire MCs with respect to Pfam annotations by computing their average over all of the MC cluster’s DA members. We denote these averages as $F_{red}$ and $F_{ext}$.

### Clustering of proteins from the PUA clan

Starting from the PUA_UR50 query dataset (see “Methods” section), our clustering method produces 71 MCs in total (Additional file 1: Fig. S1 for the MC size distribution). We find 19 MCs mapping to PUA families (Table 1) and 52 mapping to PUA associated families (Table 2). As previously mentioned, MCs can represent single or multi-family architectures and their DAs may or may not contain PUA clan families. Also, different MCs can map to the same Pfam family or architecture.

**Table 1:**
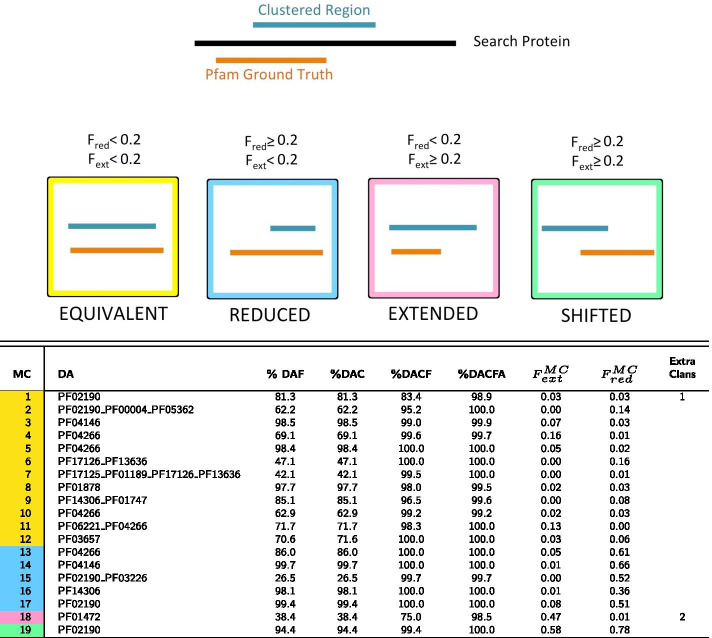
DA annotation of PUA_UR50 MCs containing PUA families

Top panel: pictorial representation of how MCs are qualitatively classified based on the overlap between DA and DA members (additionally see “Methods” section for the definition of these categories). In the table, for each MC, we report: the family-level Pfam Dominant ground truth Architecture (DA); the percentage of members featuring a DA annotation either at the family (%DAF) or at the clan (%DAC) level, these are what we call DA members; %DAC plus the percentage of members lacking one or more of the DA clans but having no additional clan’s annotation (%DACF); %DACF plus the percentage of members having clans outside of the DA but at least one DA clan (%DACFA); for DA members, the average extent of the overlap with the DA, $F_{ext}^{MC}$, $F_{red}^{MC}$; the number of extra clans that feature in %DACFA (only those present in at least 5% of clan members). MCs are colored according to the overlap between DA members and DA annotation: equivalent (yellow), reduced (blue), extended (pink) and shifted (green)

**Table 2:**
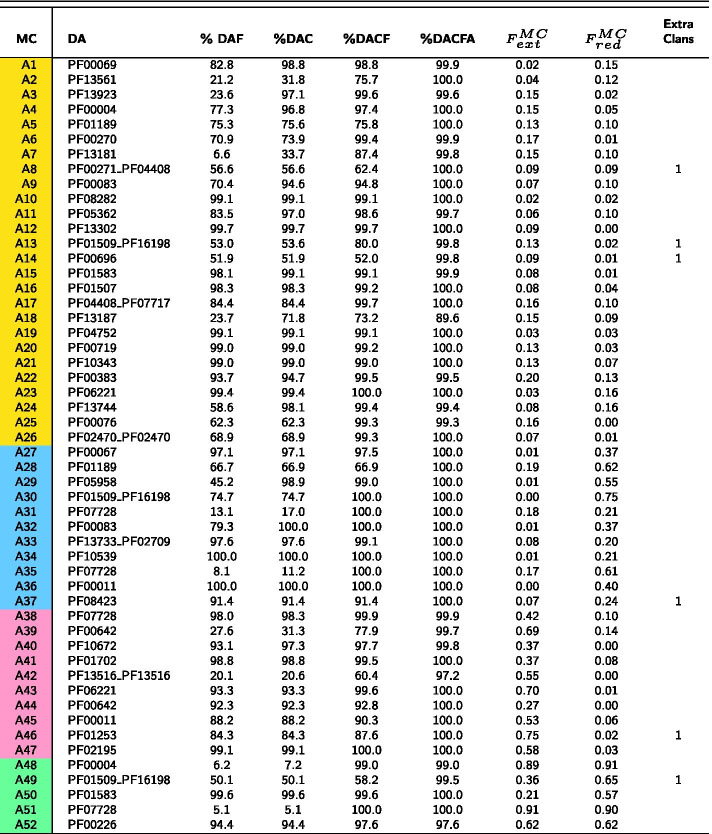
DA annotation of PUA_UR50 MCs containing PUA associated families

MCs are labeled with “A” prefix (as “associated”) (see Table 1)

#### Evolutionary consistency of MCs

**Fig. 2:**
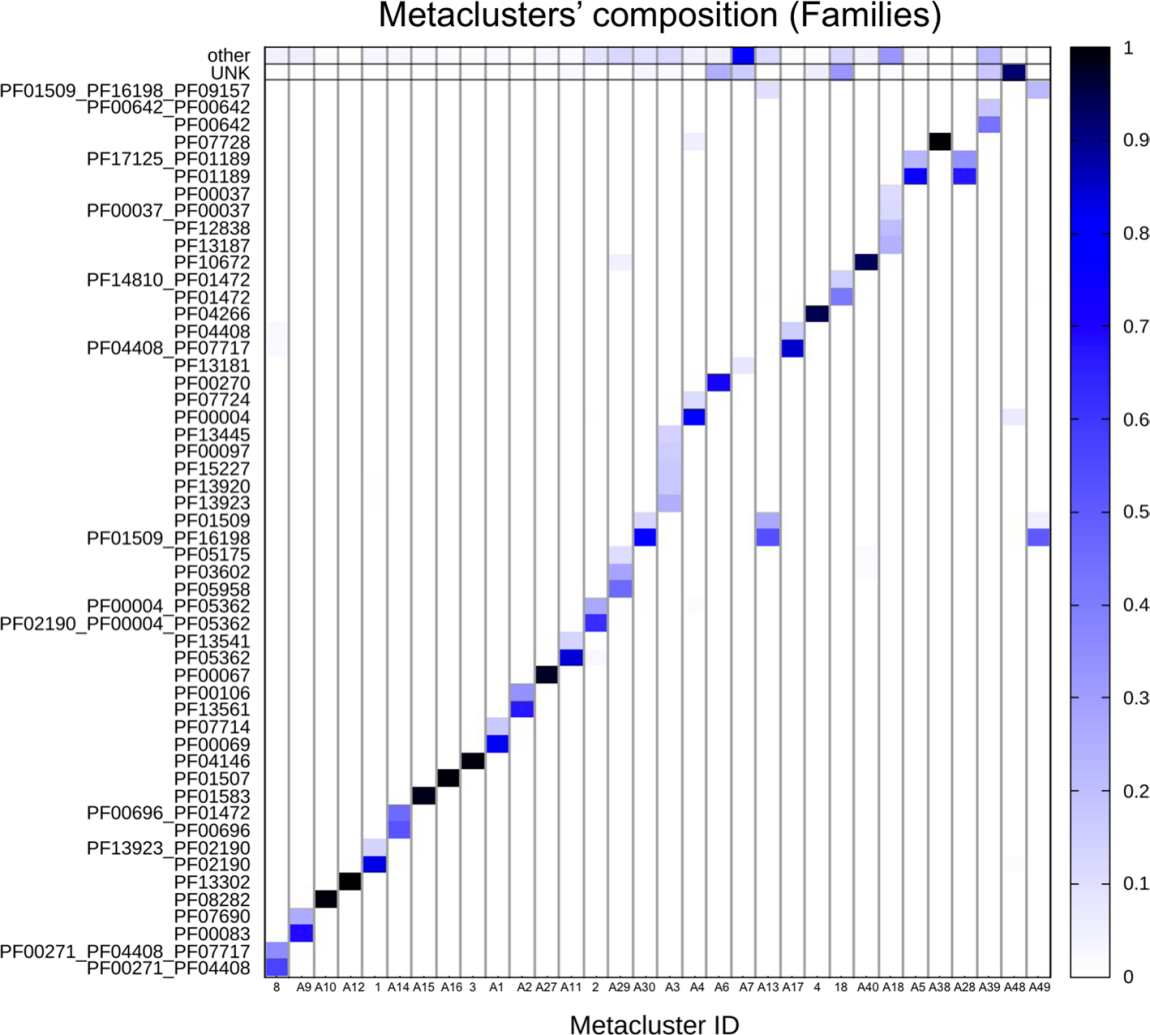
PUA_UR50 MCs versus Pfam annotation. On the *x*-axis, we list the 32 MCs (both PUA and non-PUA) with more than 500 member regions; on the *y*-axis we list the Pfam GTAs (family level) represented in each MC. We report only GTAs mapping to at least 10% of MC members and we aggregate all the remaining ones under the label “other”; finally, we label “UNK” MC members with no Pfam annotation. The heatmap is colored according to GTA fraction

The first question we address is whether DPC-generated MCs are evolutionarily consistent. In other words, we ask if MCs are formed of member sequences that share a core homologous region and could thus potentially be used as seeds for building protein families. In Tables 1 and 2 we report the percentage of member regions with a GTA that matches exactly (%DAF - family level and %DAC—clan level) or partially (%DACF and %DACFA) the DA of the cluster. %DAF or %DAC close to 100% indicate that, according to Pfam, most member sequences share a homologous core region that covers all families or clans in the DA. For example, 99.7% of 1795 MC-A12_PUA member regions are annotated in Pfam as Acetyltransf_3 (PF13302). Overall, 43.7% of MCs have %DAC > 95%. Differences between %DAF and %DAC can tell us to which extent member sequences are spread out across multiple families pertaining to the clan(s) represented in the DA. The number of Pfam families and clans and their relative weight within an MC can be better appreciated from the graphical representation in Fig. 2 (for MCs with > 500 members). For instance, MC-A3_PUA maps to several different families within the RING (CL0229) clan. This is not surprising given that the Pfam evolutionary profiles of zinc finger families within the RING clan tend to overlap (see e.g. the E-values of the families’ profile-profile alignments in the clan’s “Relationships” tab on the Pfam webserver).

**Fig. 3:**
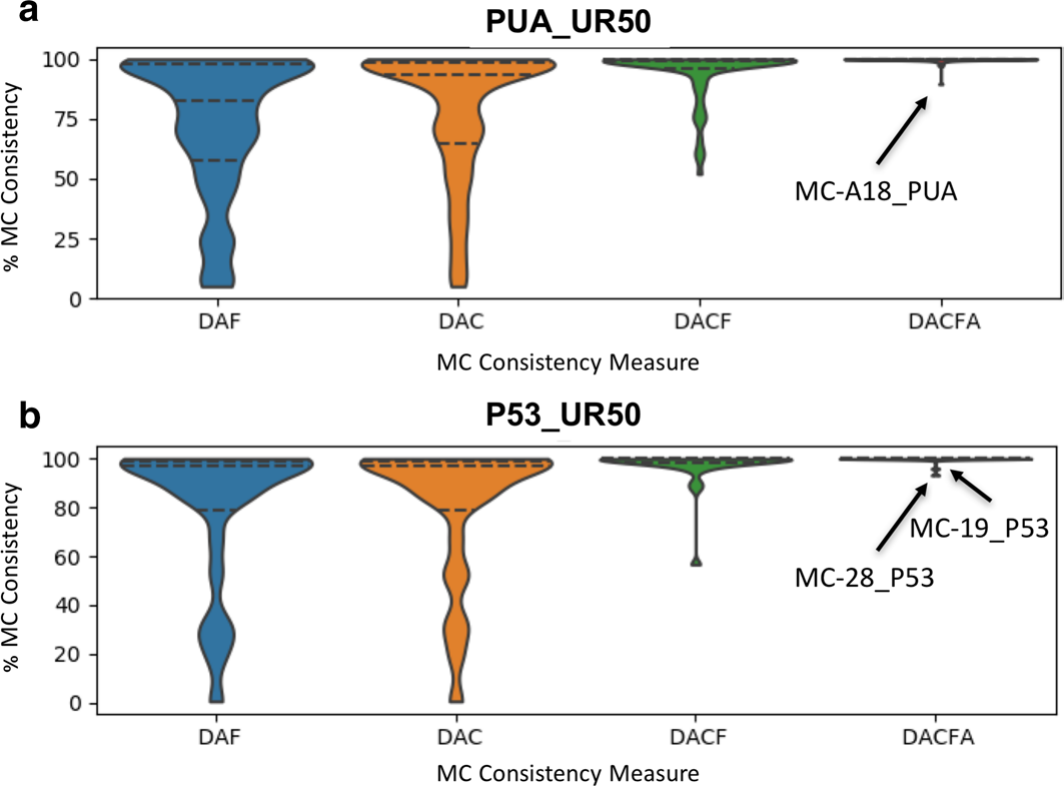
Violin plots of the distribution of %DAF, %DAC, %DACF and %DACFA. See section *Comparing metaclusters with the Pfam “ground truth”* for definitions. **a** MCs generated from the PUA_UR50 dataset, **b** MCs generated from the P53_UR50 dataset. Violin plots are such that their width is proportional to the fraction of MCs with a given value of the respective consistency measure. We label outlier MCs for %DACFA

When we add to %DAC all those members with a GTA matching only partially the DA of the MC (%DACF and, finally, %DACFA) we achieve close to full coverage in most MCs. Indeed, only one MC (MC-A18_PUA) has %DACFA < 90 (Fig. 3 and, again, Table 2).

Large percentage increases in the %DACF and %DACFA columns can point to MCs with the potential to increase coverage of existing Pfam families or clans. For example, metaclusters MC-A6_PUA and MC-4_PUA feature rather large increases in %DACF (25.7% and 30.5%, respectively). Given that the DA of these MCs is single-domain, such increases correspond to the percentage of member regions lacking any annotation in Pfam. MC-A6_PUA DA is composed of the helicase family DEAD (PF00270). Unannotated MC-A6_PUA member regions are almost always found at the N-terminus of proteins with one or more families in the Helicase_C + HA2 + OB_NTP_bind architecture. Since this is a common Pfam architecture for the DEAD domain, unannotated regions in MC-A6_PUA are likely to represent yet unrecognized members of the DEAD family. The DA of MC-4_PUA, instead, corresponds to the ASCH domain (PUA clan), with about 69% of member regions carrying this Pfam annotation. While the vast majority of remaining regions are not annotated in Pfam, in InterPro many carry an ASCH/PUA-related annotation. A closer examination reveals that MC-4_PUA is constituted of regions that are part of the “ASC-1 proper family”, as defined in the work by Iyer et al. [28], in which ASCH domains were defined for the first time. The “ASC-1 proper family” was there characterized as having a long insertion between the 3rd and 4th strand of the ASCH fold. Now that structures are available for this particular ASCH subfamily, we can additionally recognize that the domain as originally defined was cut slightly short at the C-terminus, excluding a final, extra strand and short alpha-helix (see Fig. S2 in Additional file 1). The presence of PDB structures for the C-terminal ASC-1 domain of human activating signal cointegrator 1 protein (e.g. 2E5O, 5Y7D) allowed us to build an alignment covering the whole structural domain that the family represents. Using this alignment to build a profile-HMM and running it against the Reference Proteomes database appears to capture a good number of yet unannotated regions. A large increase in %DACFA can similarly be a sign of an incomplete Pfam annotation for members of the families in the DA. One example is likely to be MC-A8_PUA, in which several member regions are likely to lack annotation for the C-terminal domain OB_NTP_bind - PF07717.

**Fig. 4:**
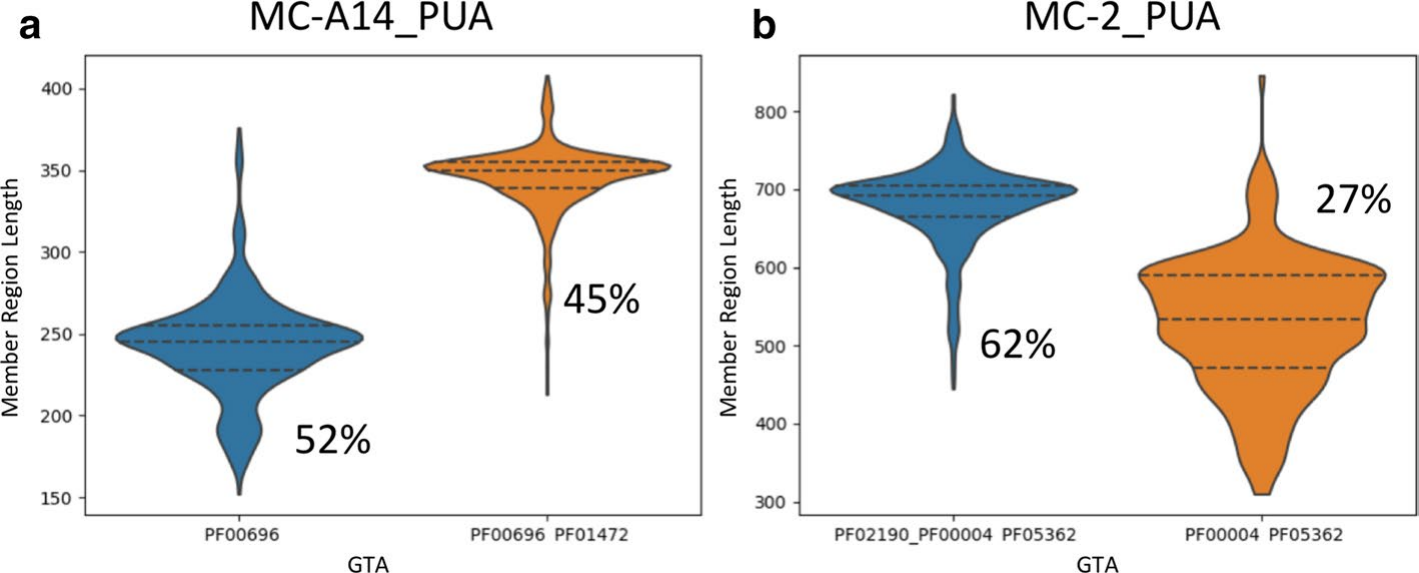
Distribution of member regions’ length for MC-A14_PUA (**a**) and MC-2_PUA (**b**). For each plot, we show the distribution of lengths of DA regions (i.e. matching the DA exactly) (blue) and, additionally, of those matching the second most abundant GTA in the MC (orange); we report the percentage of members with a given architecture next to each violin plot (note that percentages need not sum to 100%)

In other instances, percentage increases in the DACF and DACFA columns are not due to incomplete Pfam annotation but rather to the presence of subgroups of MC members featuring radically different lengths. Two such examples are MC-A14_PUA and MC-2_PUA (Fig. 4a, b). In these cases, differences in annotation between members could be easily resolved, for example, by trimming the respective MSA alignments to the shortest lengths. Further, there are cases in which the DA does not provide an accurate description of the annotation of the MC. This happens when a family has only a marginal overlap with a number of member regions and absolutely no overlap with others. In this case, we can have large increases in %DACF or %DACFA that are artifacts of the way we annotate the GTA of MC members.

One example is MC-A28_PUA where about 33% of member regions overlap with a small portion of family PF17125, which is located at the N-terminus and is not part of the DA of the metacluster. We note that in principle it would be possible for members counted as part of %DACFA to map to completely different, non-overlapping sections of the DA. These would be regions that are not homologous to each other. During our analysis of the PUA clan and associated families, however, we did not come across any such example, suggesting that these are unlikely to be common occurrences.

**Fig. 5:**
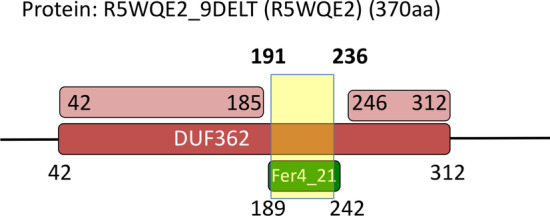
Example (protein R5WQE2) of nesting of an MC-A18_PUA region into a family of the “DUF362-like superfamily”—CL0471 clan. Solid-colored rectangles mark Pfam family annotations. The light red rectangle shows the actual region of R5WQE2 that aligns to the DUF362 profile-HMM. Note that in this specific case, even in the Pfam annotation nesting of Fer4_21 into DUF362 is not accounted for, resulting in two overlapping Pfam annotations. The yellow box marks a hit obtained using the profile-HMM derived from MC-A18_PUA

A quick look at Tables 1 and 2 reveals a couple of outstanding cases among all MCs produced. First MC-A18_PUA, which is by far the metacluster with the lowest %DACFA (89.6%). This indicates that > 10% of member regions carry Pfam annotation that appears to be incompatible with the DA of the MC; in other words, these regions would appear to be evolutionary unrelated to the others. The metacluster’s DA is constituted of Pfam family Fer4_9 (PF13187), which itself is part of the 4Fe-4S (CL0344) clan. Most families in this clan represent iron-sulfur cluster binding motifs (Fe-S BMs) characterized by a CCxxxC signature and they often feature two consecutive copies of such motif, PF13187 being one of them. A more than 45% increase in member coverage from %DAF to %DAC for MC-A18_PUA indicates that Pfam annotation of MC members covers other families of the 4Fe-4S clan. Examples are members annotated as part of the double-motif families Fer4_7 (PF12838) and Fer4_10 (PF13237) as well as those annotated with two copies of the single motif family Fer4 (PF00037). All of the above are families with close evolutionary relationships within the clan. There is, however, a fraction of members that are annotated as belonging to families such as Radical_SAM (PF04055) and DUF362 (PF04015) that are found in clans other than CL0244. What is happening in these cases, however, is that the MC members span Fe-S BM regions that are nested within these longer domains. As mentioned above, our in-house Pfam annotation protocol does not take into consideration nested domains. If family A spans region a to b of a protein and family B the region a’ to b’ of the same protein with a’> a and b’ < b, region a’-b’ is assigned to one family only, the one with the lowest E-value, which will generally belong to the longest family. This is what happens for some of the MC-A18_PUA members, whereby regions that in Pfam are annotated as Fe-S BMs are instead annotated by our protocol as belonging to the family the BMs are nested within; we show one example of this in Fig. 5. In conclusion, we can say that the vast majority of MC-A18_PUA member sequences consistently represent regions spanning Fe-S BMs.

**Fig. 6:**
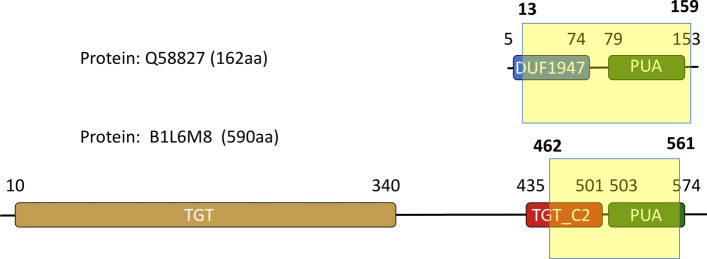
Annotation for protein Q68827 and B1L6M8. **a** Top: Pfam annotation for Q68827; the yellow box indicates a hit obtained using profile-HMMs derived from the metacluster MC-18_PUA. Bottom: Pfam annotation of protein B1L6M8; the yellow box indicates a hit obtained using profile-HMMs derived from the metacluster MC-18_PUA

A second outstanding case is represented by MC-18_PUA. This metacluster has a significant number of member regions that feature extra clans not part of the DA represented by the PUA (PF01472) family (> 20% increase in %DACFA). While other clans have even larger %DACFA increases, MC-18_PUA is unique in that it is the only one featuring two extra non-DA clans (last column in Table 1).This would not constitute a problem if the clans were added sequentially along the sequence but could be problematic if the two clans were found in a similar position upstream or downstream of the DA in different MC members. For this reason, MC-18_PUA needs to be analysed in detail. We start by observing that $F_{ext}^{MC}=0.47$, indicating that even DA members typically extend well beyond their PUA domain  and into a (in this case N-terminal) region not annotated by Pfam. A number of other MC members, however, feature additional Pfam annotation at the N-terminus of the PUA domain: 13.6% feature a TGT_C2 (PF14810) domain, 8.4% a DUF1947 (PF09183) domain and, finally, 1% a TruB_C_2 (PF16198) domain. When present, these families are well covered by the MC-18_PUA member sequences: 97% of TGT_C2 amino acids are covered, 67% of DUF1947 and 61% of TruB_C_2, respectively. Worryingly, these three families are not found in the same Pfam clan, that is, they are not recognized as homologous by the Pfam classification: DUF1947 is part of the pre-PUA (CL0668) clan that, as the name indicates, is constituted of regions that are found N-terminal to PUA domains, TruB_C_2 is part of the PseudoU_synth (CL0649) clan and, finally, TGT_C2 is not part of any Pfam clan. We notice, however, that TGT_C2 regions are almost always found N-terminal to PUA domains; more importantly, alignment between representative structures of TGT_C2 and DUF1947 reveals striking similarities (see Fig. 6 and Additional file 1: Fig. S3) thus suggesting a common evolutionary origin for the two families. TGT_C2 would then represent a novel pre-PUA domain to be added to the Pfam clan of the same name. Interestingly, even a very sensitive profile-profile alignment method such as HHpred [29] appears not to be able to find a relationship between TGT_C2 and pre-PUA. In particular, when we ran HHpred using the Pfam seed multiple sequence  of family TGT_C2 against Pfam v33.1 we found no significant match to any of the pre-PUA clan families. The other extra family found in about 1% of MC-18_PUA member regions, TruB_C_2, is instead structurally (thus evolutionarily) unrelated to both DUF1947 and TGT_C2. Indeed, most MC-18_PUA alignments that feature TruB_C_2 have E-values of borderline significance (> 0.01) further supporting the notion that these are likely to represent noise.

In summary, analysis performed using Pfam annotation suggests that the vast majority of MCs are evolutionarily sound with member sequences that share between them a core homologous region. This core region may correspond to the DA of the metacluster or be longer/shorter as we will discuss more in detail in the following.

**Table 3:** Member regions' statistics for PUA_UR50 MCs

MC (PUA)	Size	Average length	SDL	LC fraction	MC (PUA)	Size	Average length	SDL	LC fraction
*1*	1575	207.4	42.5	0.04	*11*	120	396.3	*61.8	0.04
*2*	862	623.1	*102.9	0.04	*12*	109	154.2	26.7	0.02
*3*	791	152.7	30.5	0.02	*13*	430	40.6	3.3	0.00
*4*	682	125.1	16.9	0.01	*14*	399	54.2	3.1	0.00
*5*	487	119.7	10.9	0.02	*15*	309	125.9	35.5	0.01
*6*	452	109.8	14.4	0.02	*16*	162	95.5	11.1	0.02
*7*	432	441.4	44.8	0.03	*17*	162	115.8	15.5	0.05
*8*	392	136.7	19.9	0.02	*18*	675	148.5	24.0	0.02
*9*	282	320.7	48.7	0.02	*19*	339	88.4	13.3	0.07
*10*	251	102.3	8.7	0.02					

MC (A-PUA)	Size	Average length	SDL	LC fraction	MC (A-PUA)	Size	Average length	SDL	LC fraction
*A1*	69369	223.7	29.9	0.02	*A28*	1365	83.9	13.2	0.02
*A2*	8908	203.9	19.1	0.05	*A29*	615	84.5	8.4	0.04
*A3*	8324	49.1	5.6	0.00	*A30*	506	47.7	4.8	0.01
*A4*	4523	158.6	36.5	0.02	*A31*	464	99.5	10.8	0.01
*A5*	3559	210.2	28.7	0.03	*A32*	406	191.7	18.6	0.06
*A6*	3386	193.1	17.9	0.05	*A33*	340	183.3	33.4	0.02
*A7*	2934	102.7	12.1	0.02	*A34*	294	99.0	10.6	0.03
*A8*	2915	347.2	*76.5	0.04	*A35*	285	48.8	6.6	0.01
*A9*	2870	392.0	48.9	0.06	*A36*	248	59.6	7.5	0.03
*A10*	2735	257.3	12.6	0.02	*A37*	198	210.5	30.9	0.03
*A11*	2392	146.5	40.2	0.03	*A38*	1588	226.5	45.2	0.01
*A12*	1795	153.3	11.4	0.01	*A39*	691	86.7	19.7	0.00
*A13*	1751	235.9	25.9	0.03	*A40*	565	369.6	35.2	0.04
*A14*	986	289.8	*57.4	0.05	*A41*	430	339.6	36.9	0.01
*A15*	851	164.1	13.9	0.03	*A42*	359	328.7	*52.0	0.03
*A16*	839	193.3	26.1	0.01	*A43*	267	165.1	32.6	0.08
*A17*	700	259.4	29.3	0.03	*A44*	208	36.3	3.6	0.00
*A18*	556	46.8	5.0	0.02	*A45*	186	181.8	37.9	0.09
*A19*	452	173.0	17.0	0.02	*A46*	121	311.9	26.4	0.04
*A20*	384	189.3	32.3	0.01	*A47*	110	211.3	23.1	0.02
*A21*	193	293.9	36.0	0.02	*A48*	677	87.5	15.4	0.02
*A22*	190	114.7	13.7	0.02	*A49*	625	119.5	19.9	0.03
*A23*	172	43.0	4.2	0.00	*A50*	277	77.9	6.4	0.01
*A24*	162	60.4	4.8	0.01	*A51*	178	132.0	15.4	0.01
*A25*	146	86.6	16.1	0.04	*A52*	126	62.2	10.2	0.11
*A26*	135	216.7	12.3	0.02					
*A27*	3181	196.9	21.1	0.02					

Top section: MCs containing PUA domains; bottom section, MCs containing PUA-associated domains (A-PUA, with “A” prefix). For each MC, we report size (i.e., number of sequence members), average and standard deviation of members’ lengths and, the fraction of residues (of all members) that are found in low-complexity regions (LC fraction, using the segmask software of the NCBI-BLAST+ suite [30]). We flag MCs (*) for which the SDL is larger than 50 amino acids (about the size of a small domain)

#### Comparison between MCs and Pfam families boundaries

Another important aspect of comparing two protein classifications entails investigating by how much the boundaries of the respective clusters or families differ when evaluated on the same sequences. The quantities $F_{MC}^{ext}$ and $F_{MC}^{red}$ in Tables 1 and 2 indicate the extent of the agreement between the boundaries of DA members and the respective Pfam annotations (these are averages over all DA members, as explained in previous section). To provide some qualitative insight, we classify MCs into the following four categories according to the agreement of their DA members with the DA Pfam family boundaries (see inset figure in Table 1): equivalent (both $F_{ext}^{MC}$ and $F_{red}^{MC}$ $<0.2$, yellow); reduced ($F_{ext}^{MC}<0.2$ and $F_{red}^{MC}\ge 0.2$, blue), extended ($F_{ext}^{MC}\ge 0.2$ and $F_{red}^{MC}<0.2$, pink) and, finally, shifted (both $F_{ext}^{MC}$ and $F_{red}^{MC}$ are $\ge 0.2$, green). Equivalent MCs are the closest to the DA architectures in terms of their boundaries; the other categories feature cases that may be worthy of further inspection. MC-A29_PUA, for example, features member regions that typically cover only about half of the DA family tRNA (Uracil-5-)-methyltransferase (PF05958) as annotated by Pfam in the full-length proteins they belong to. Structural data indicate that, in fact, Pfam family PF05958 covers two structural domains: a so-called central domain, which hosts a [Fe4S4] cluster, and a catalytic domain typical of SAM-dependent methyltransferases. MC-A29_PUA covers only the catalytic domain of the tRNA (Uracil-5-)-methyltransferase, albeit imperfectly (see Additional file 1: Fig. S4). In another example, the MC-18_PUA($F_{ext}^{MC}=0.47$) metacluster we already discussed, the ’true’ DA is likely to be constituted of the double-domain architecture pre-PUA+PUA rather than by PUA only.

Interesting cases are constituted by MC-1_PUA and MC-17_PUA, both mapping to the Lon_substr_Bdg family (PF02190). While the first MC has “equivalent” status, the second one is a “reduced” MC mapping only to half of the domain. However, the Lon_Substr_bdg domain contains two structural units (see Additional file 1: Fig. S5), of which MC-17_PUA captures only the first.

When discussing boundaries, we should not forget that some MCs feature a bi-modal distribution of their members’ lengths (see for example Fig. 4a, b). In these cases, the average measures $F_{ext}$ and $F_{red}$ cannot capture the full complexity of boundary differences with respect to the Pfam annotation.

#### MCs with minimal Pfam annotation

Some MCs with single-family DA feature low %DAC and high %DACF indicating that, for the most part, they are constituted of member sequences that are devoid of any Pfam annotation; among these are MC-A7_PUA, MC-A39_PUA and MC-A48_PUA. MC-A48_PUA member regions, 92% of which are unannotated, are found in ATP-dependent Lon protease proteins and typically cover a helical region located at the N-terminus of the AAA (PF00004) ATPase domain (Additional file 1: Fig. S6). This region could potentially be built into a short “pre-AAA” motif. MC-A7_PUA (23% of unannotated members, see Fig. 3) and MC-A39_PUA (46.5%) map, respectively, to tetratricopeptide-like repeats or TPRs (CL0020) and Cys3His zinc-binding domains (CL0537) also often found in tandem repeats. Tandem repeats such as these, which are relatively short and often feature a high degree of divergence in sequence, are notoriously difficult to classify exhaustively. It is thus not surprising that many elements of these MCs do not carry annotation in Pfam. Note that increases of %DACFA in these two MCs are mostly due to the presence of members with a higher number of repeated domains than found in the DA. There might be scope for using these MCs as a basis to boost coverage of the respective clans.

#### Degeneracy of MCs with respect to Pfam families

**Fig. 7:**
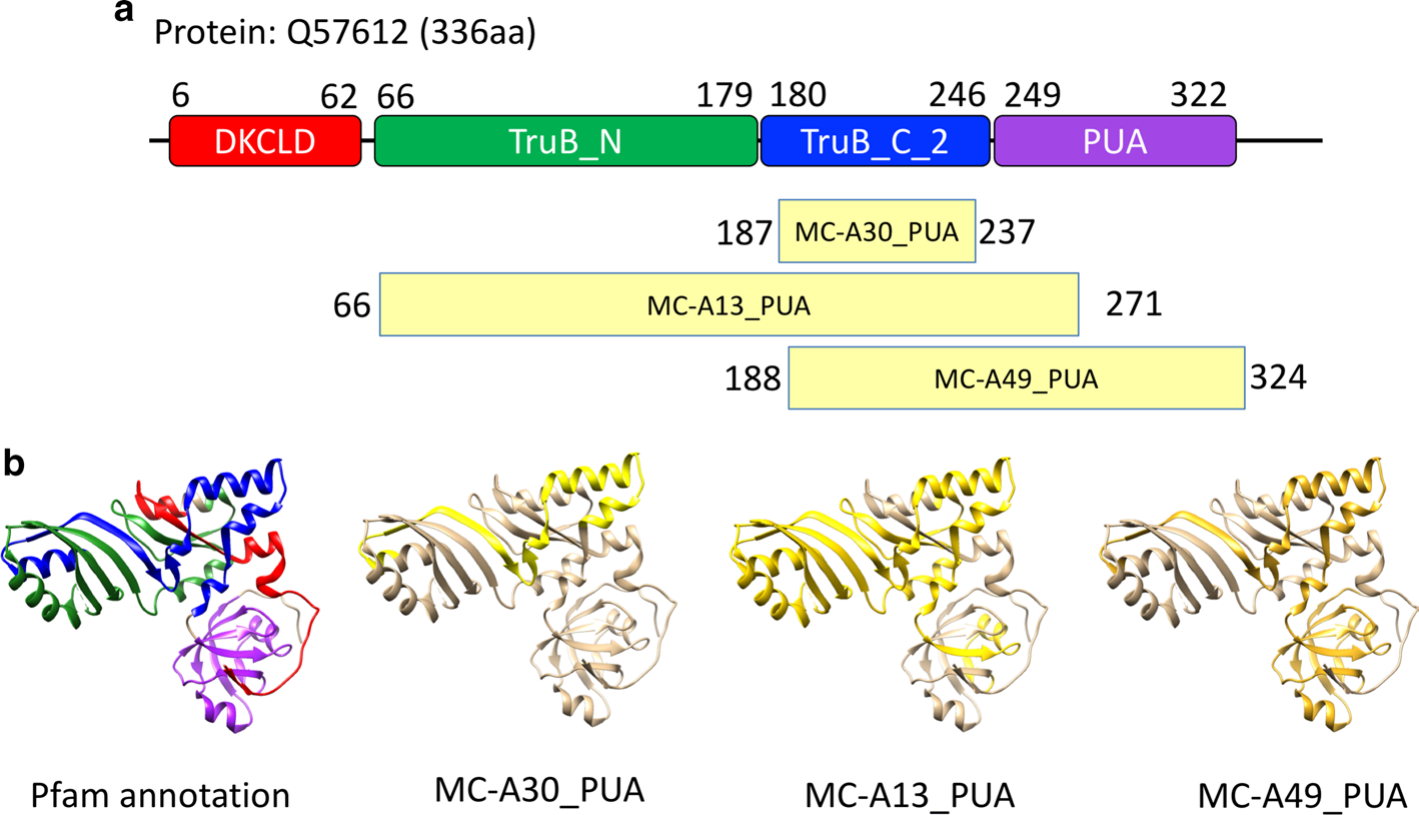
Annotation for protein Q57612 (2apo in pdb). **a** Sequence with Pfam annotation; yellow boxes shows the hits obtained using profile-HMMs derived from the metaclusters MC-A30_PUA, MC-A13_PUA and MCA-49_PUA. **b** Structure of 2apo PDB chain A, colored following Pfam classification in panel A and according to the matches with the profile-HMMs of MC-A30_PUA (aa 187-237) in yellow, MC-A13_PUA (aa 66-271) in gold and MC-A49_PUA (aa 188-324) in dark gold

In some instances, DPC produces multiple clusters that map to the same Pfam family or group of families. Here it is worth pointing out that we use the DPC algorithm to cluster alignments rather than protein sequences. This means that alignments of the same protein region to different proteins are treated as separate entities. Our clustering protocol tries to ensures that when two regions of the same protein of about the same size have a large overlap, they are classified as belonging to the same cluster. For overlaps that are small with respect to the length of the alignments being compared, the regions may end up in different MCs. One such example is represented by the trio of clusters MC-A30_PUA, MC-A13_PUA and MC-A49_PUA, all of which feature the same DA, namely, TruB_N + TruB_C_2 (PF01509+PF16198). Both of these Pfam families are part of the PseudoU_synth (CL0649) clan. In Fig. 7, taking as sample sequence one for which a structure is available, we show that although the 3 clusters share the same DA, the actual set of families they cover is quite different. In fact, the three MCs belongs to three different boundary categories (see Table 2): reduced (MC-A30_PUA), equivalent (MC-A13_PUA) and shifted (MC-A49_PUA). Contrary to MC-A13_PUA, that truly corresponds to the DA families, MC-A30_PUA covers mainly TruB_C_2 with minimum overlap to the first family, and MC-A49_PUA covers mainly TruB_C_2, but also extends beyond it in a region that when annotated is reported to be part of a PUA-clan family.

In the Pfam clan, the pseudouridine synthase domain has sometimes been split into two families (TruB_N +TruB_C_2, PseudoU_synth_1x2, PseudoU_synth_1+DUF2344) or otherwise classified as a single family (PseudoU_synth_2, TruD). The difficulty for a consistent evolutionary classification of this domain comes primarily from two things: (1) the pseudouridine synthase domain appears to be formed by a tandem duplication the two moieties of which share often very little sequence similarity with each other (and only structural similarity in terms of their general topology) and (2) the two homologous moieties feature strand swapping and sometimes nesting of additional domains. The latter is the case for sequences in the TruD family, which in Pfam additionally covers a nested domain that should instead be built as a separate family outside of the CL0649 clan (see Additional file 1: Fig. S7). Also, the boundaries of paired families such as TruB_N and TruB_C_2 do not seem to reflect the structural organization of the duplication very well (see red and blue regions in Additional file 1: Fig. S8). Indeed, the current boundaries of the two families represent regions of very different structure, with the TruB_C_2 open and elongated structure not reminiscent of a typical structured domain. There is, for example, no pairwise structural alignment produced by DALI with default settings for the TruB_N and TruB_C_2 Pfam annotated regions of PDB structure 3u28 A. We suggest that building a family covering the entire pseudouridine synthase domain would also in this case (as in, for example, PseudoU_synth_2) be the best option. Finally, the Pfam nomenclature of families that map to tRNApseudouridine synthase B proteins is quite confusing. TruB_N is the N-terminal part of a PseudoU_synth domain, TruB_C is a PUA domain, TruB_C_2 is the C-terminal part of a PseudoU_synth domain and TruB-C_2 is again a PUA domain. Although we understand family names have a historical relevance, a rethinking of this particular set of names may be beneficial. It is interesting to note that in our automatic classification the N-terminal boundary of the TruB_C_2 family is well matched by both MC-A30_PUA and MC-A49_PUA, highlighting the differences between the two moieties of the pseudouridine synthase domain.

#### Coverage of the PUA clan by DPC-generated MCs

**Fig. 8:**
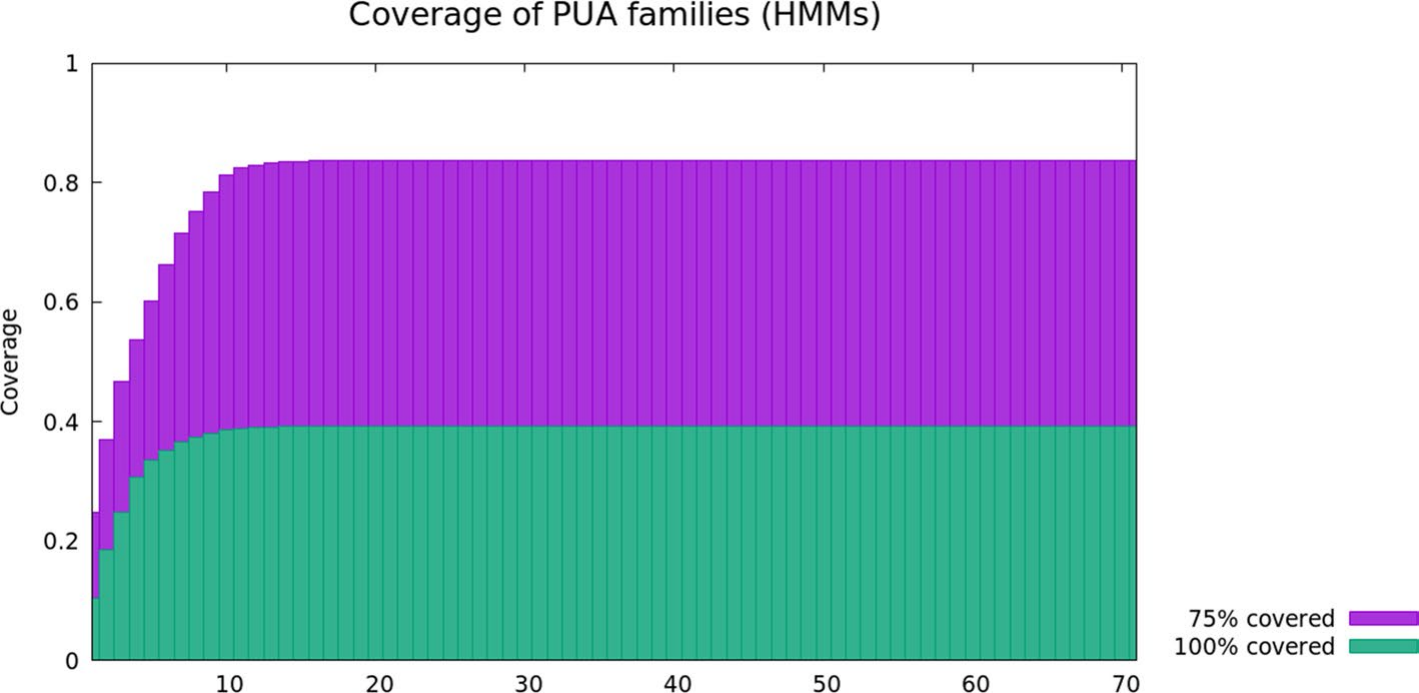
Coverage computed on proteins in UniRef50 including at least a region of the PUA clan according to Pfam-A. By running the profile-HMMs derived from the metaclusters, we search for hits covering at least 75% or 100% of a PUA region. The graph shows the fraction of the PUA clan covered using an increasing number of metaclusters. After after the 15th metacluster, the fraction does not improve, because of some redundancy in PUA MCS, which are 19. Also, MCs appearing after the 19th do not map to a PUA clan family, and do not contribute to any increase in the coverage

So far, we have looked at how consistent the Pfam annotations are within the DPC-generated MCs (in other words, we looked at the accuracy of our classification). Clearly, it is also important to know to what extent the automatically-generated classification recapitulates Pfam’s coverage of the sequence space. In this section, we investigate coverage of PUA clan regions within the UniRef50 database: we consider all regions that produce significant alignments to the MCs-derived profile-HMMs (hmmsearch run against PUA_UR50, sequence E-value < 0.01, Hit E-value < 0.03). We say that a MC covers a PUA region if there is at least one of the profile-HMM hits covering >= 75% or = 100% of it. We plot the cumulative coverage of the Pfam PUA clan when ranking MCs from the one that contributes the highest coverage to the one that contributes the lowest coverage (Fig. 8); we note that proteins are counted only once, even if covered by more than one MC. It is interesting to see that coverage converges after a number of MCs that is roughly equivalent to the number of Pfam families in the PUA clan (11 total). We see that > 80% of the PUA clan regions are covered for at least 75% of their length by the top 15 clusters. While a fraction of Pfam regions in the PUA clan is not covered by MCs, we should point out that most PUA-covering MCs include at least some additional regions not currently annotated in Pfam, which are likely to represent new clan members. The flattening out of the curves that we observe after 10–15 MCs reflects the fact that most of our 71 MCs cover PUA-associated families rather than PUA families.

### Clustering of proteins from the P53-like clan

**Fig. 9:**
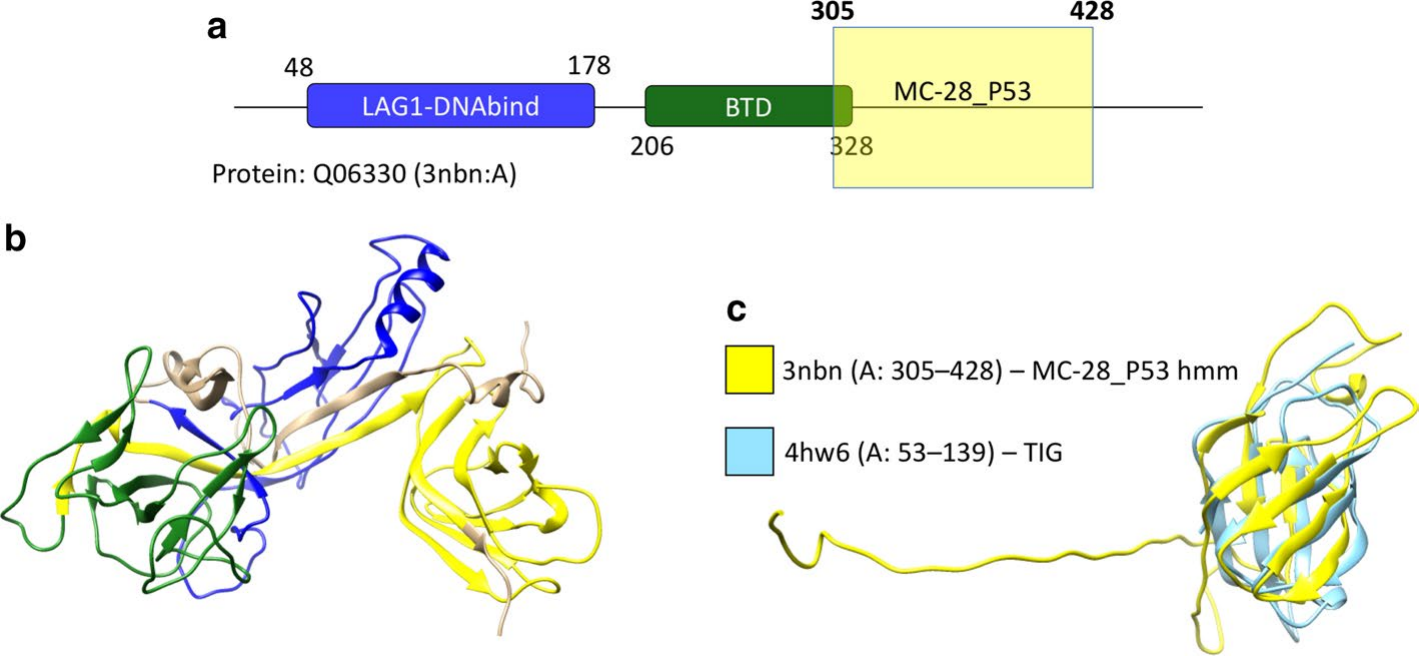
Annotation for protein Q06330. **a** Sequence Pfam annotation; the yellow box marks a hit obtained using the profile-HMM derived from metacluster MC-28_P53. **b** Pfam and MC-28_P53 annotations of **a** mapped to one of the available structures of Q06330 (PDBid 3nbn:A). Color code for families and regions is the same as in (A). **c** Structural alignment between the MC-28_P53’s annotated region of 3nbn (yellow) and the TIG domain of PDB structure 4hw6 (light blue). Alignment obtained with DALI pairwise online tool; alignment features: Z = 6.2, RMSD = 2.2, percent sequence identity = 25

Clustering of the PUA clan, which is described in detail in the previous sections, has uncovered several interesting features of the relationships between the Pfam families involved. Our clustering procedure utilizes few adjustable parameters (“Methods” section) and we did not perform any systematic exploration of the parameter space. Rather, parameters were mostly chosen following heuristic rules from the literature, thus considerably limiting the risk of over-fitting. Nevertheless, we did use the PUA clan to tune some aspects of our procedure (e.g. thresholds for merging MCs). As a consequence, in this section we report on results obtained when running DPC-based clustering on a second Pfam clan, when all parameters have been left unchanged with respect to the ones used for PUA. This should provide additional evidence of the fact that our method could be successfully extended to the analysis of larger portions of the sequence space. In particular, we run our DPC procedure on the P53-like clan (Tables 4, 5). Overall, the results appear to be in line with the ones obtained for PUA. Our procedure generates 28 MCs of size > 100, of which 53.6% have %DAC > 95%. Only two MCs, MC-19_P53 and MC-28_P53, have %DACFA < 98%. MC-19_P53 is peculiar in that the vast majority of its members lack Pfam annotation (+ 95.4% in the %DACF column with respect to the single-domain DA). This may be explained by the high value of the low-complexity residue fraction in this MC ($LC=0.58$, Table 4), suggesting that its member regions are unlikely to represent a structural domain. Additionally, low-complexity regions are more likely to align to non-homologous sequences (thus potentially explaning %DACFA = 95.9%). MC-28_P53 contains 132 sequences, 54% of which are not annotated, 33% annotated as PF09270 (BTD), 7% annotated as PF01833 (TIG) and, finally, 5% annotated as BTD + TIG. BTD is not a P53-like family, however, it is found by our clustering algorithm because BTD is commonly located at the C-terminus of the P53-like LAG1-DNAbind family. Although the BTD annotation is the most present in MC-28_P53, the domain it represents is poorly covered. Indeed, only a few amino-acids at the C-terminus of BTD are found in MC-28_P53 members. On the contrary, when present, TIG regions are well covered. Searching the Reference Proteome dataset with a MC-28_P53 generated profile-HMM we found 2083 significant hits (hmmsearch, sequence E-value < 0.01, Hit E-value < 0.03). About half of these mapped to TIG domains, while the rest although often found C-terminal to a LAG1-DNAbind + BTD architecture are not annotated in Pfam. Finally, we ran MC_28-P53 profile-HMM against the PDB, finding as top matches yet unannotated regions located at the C-terminus of LAG1-DNAbind + BTD architectures (see Fig. 9a–c for an example). Of these, we focused on SUH_HUMAN (Q06330, PDBid 3nbn_A). The region of 3nbn A aligned to the MC-28_P53’s profile-HMM appears to be well-structured (Fig. 9b, yellow) and it is structurally similar to TIG domains (Fig. 9c). In conclusion, MC-28_P53 is likely to represent a TIG family covering a good number of TIG domains not yet annotated in Pfam. Coverage of Pfam P53-like clan’s regions by P53 MCs is comparable to the one observed for the PUA clan (see Additional file 1: Fig. S10).

In general, in the case of the P53 clan, we notice two main differences with respect to the clustering of the PUA clan. First, we see what appears to be a higher degree of MC redundancy with respect to the Pfam classification. For example, 6 MCs have PF00907 as their DA and 4 MCs feature PF05224 in theirs. It should be noted, however, that in the case of PF00907 only two MCs have an average length of more than 50aa. In fact, MC-14_P53 and MC-27_P53 have length < 30aa, which is much shorter than the length of the average protein domain [31]. In Additional file 1: Fig. S9 we show a graphical view of how the different MCs map to this Pfam family. Second, with respect to the PUA clan, on average, MC boundaries appear to match less well those of the DA families. Indeed, in Table 5 we observe several MCs with high $F_{ext}^{MC}$ and/or $F_{red}^{MC}$. We notice, again, that this is often the case for MCs of short average length.

**Table 4:** Member region’s statistics for P53_UR50 MCs (see Table 3)

MC	Size	Average length	SDL	LC fraction	MC	Size	Average length	SDL	LC fraction
*1*	25859	185.9	30.8	0.02	*16*	281	126.1	13.5	0.01
*2*	941	171.9	26.9	0.01	*17*	254	68.2	7.4	0.01
*3*	481	279.4	30.7	0.01	*18*	213	45.8	2.9	0.00
*4*	467	465.9	*92.3	0.01	*19*	194	111.6	15.0	0.58
*5*	462	204.5	31.0	0.02	*20*	154	39.0	3.1	0.02
*6*	231	494.3	*106.3	0.02	*21*	145	34.0	3.5	0.01
*7*	231	340.8	*52.0	0.05	*22*	9428	207.9	17.6	0.03
*8*	225	191.1	29.7	0.02	*23*	699	135.1	21.9	0.02
*9*	166	475.7	*107.6	0.02	*24*	124	399.5	*58.8	0.03
*10*	163	126.0	10.4	0.01	*25*	203	136.3	15.9	0.01
*11*	110	421.4	*53.4	0.05	*26*	158	111.1	14.2	0.01
*12*	761	67.8	8.7	0.01	*27*	137	28.2	2.6	0.00
*13*	531	43.6	4.4	0.00	*28*	132	137.7	21.0	0.05
*14*	525	29.6	2.7	0.00					
*15*	363	38.2	5.0	0.00

**Table 5:**
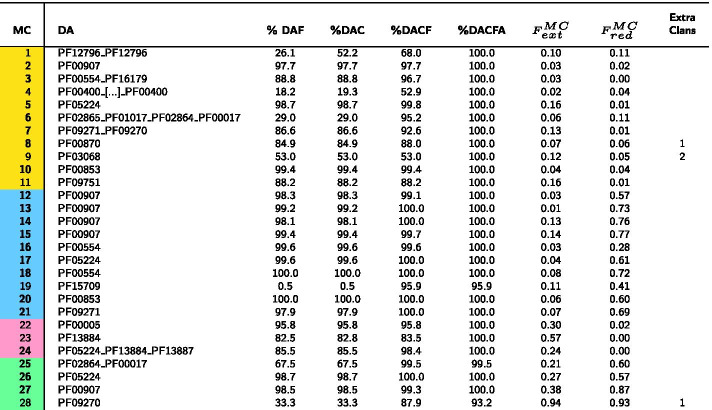
DA annotation of P53_UR50 MCs (see Table 1 for column description)

Highlighted in bold MCs contain P53 domains. DA including “[...]” represent a very long repeat, which has not been reported entirely for formatting reasons

## Discussion

Automatic classification of proteins into homologous regions or domains is a notoriously difficult problem due to the complexity of evolutionary relationships between proteins, which include but are not limited to the existence of multi-domain architectures, domain nesting and tandem repeats. Moreover, domain evolutionary divergence at the sequence level can be extremely high thus making it exceedingly difficult, if not impossible, to group into individual families all homologous regions. Finally, domain boundaries can be blurry. For these reasons, databases that attempt to classify protein families and domains use extensively either manual annotation or structural knowledge (often both). Nonetheless, unsupervised, automatic domain classication from sequence [13] [15] [19] is extremely relevant both to identify conserved regions that can later be manually refined and annotated to create novel families and for complementing manual classification in differential domain analysis of large datasets with a high degree of sequence novelty (such as for example sequences from environmental genomics [32] [33]).

Here, we have presented a new unsupervised procedure for automatic protein domain classification based on Density Peak Clustering. In the proof-of-principle experiment presented in this work, we clustered proteins that feature domains from one of two separate Pfam clans (PUA and P53-like). We showed that, in most cases, automatically-generated metaclusters (MCs) represent single or multi-domain architectures which, overall, display a good agreement with the Pfam annotation. With respect to the presence of multi-domain MCs, we should emphasize that our procedure clusters evolutionary modules (using sequence similarity) rather than directly structural domains (see definitions in [1]). Because of this, it may be difficult for our method to split into separate MCs structural domains that are only (or overwhelmingly) observed in joint architectures, unless these domains are separated by long regions of low conservation. In the two clans we have analysed, choosing a number of MCs that is roughly comparable to the number of Pfam families belonging to the clan provides good coverage of their member regions (Additional file 1: Fig. S10). We do observe, especially in the analysis of the P53-like clan, a certain degree of redundancy between MCs (i.e. multiple MCs mapping to the same Pfam family). Although it is possible that this redundancy could be significantly reduced by discarding short length MCs, this indicates that the MC-merging step of our procedure (see “Methods” section) could potentially be improved. With respect to the choice of the method’s parameters, our benchmarking experiments suggest that the clustering is robust within a certain range of variation in their values and, additionally, of the size of the starting (query) dataset (Additional file 1: Table S1).

In general, significant differences between clans exist in terms of size, evolutionary divergence, complexity of architecture and structural class of their families. Although these diversity cannot be recapitulated in full by the analysis of only two Pfam clans shown here, it is worth pointing out that our clustering experiment did extend to numerous families outside of the PUA and P53-like clans (see Tables 2, 5). This is due to the fact that our method runs on full-length sequences and that about 45% and 39% of PUA and P53-like member regions, respectively, are part of multi-domain proteins.

## Conclusions

Overall, our procedure based on Density Peak Clustering identified interesting conserved regions in the sets of proteins we analyzed, often in agreement with the Pfam classification. We have provided evidence, based on the analysis of two Pfam clans, that our method has potential for supporting manual annotation of protein families. While the method also identified a possible novel family (MC-A48_PUA), further experiments are required to assess its potential in domain discovery. In particular, it would be important to test more numerous and diverse clans and, at the same time, to additionally compare the clustering results with protein family classifications other than Pfam.

## Methods

In the following sections we describe in detail the different steps of our clustering procedure, which consist of: producing BLAST alignments of our query (clan) database against UniRef50, primary clustering of alignments falling on the same query sequence, metaclustering of primary clusters, finally merging of metaclusters.

### BLAST searches

Our database of reference throughout this work is UniRef50 (v. 07/2017). Given a Pfam clan, for example PUA, we generate a dataset constituted of all UniRef50 full-length sequences that carry a PUA clan member annotation by matching their UniProtKB ids with those of sequences in Pfam-A.full v.31. The dataset obtained is then named PUA_UR50, and contains 4083 protein sequences. With the same procedure we obtain P53_UR50, containing 2022 protein sequences. Next, for each sequence (query) in the dataset, we perform a local alignment search against the full UniRef50 database using NCBI BLAST (v. 2.2.30+)[23] and save all alignments with E-value < 0.1 (up to 5 millions, using the max_target_seqs option of BLAST).

**Fig. 10:**
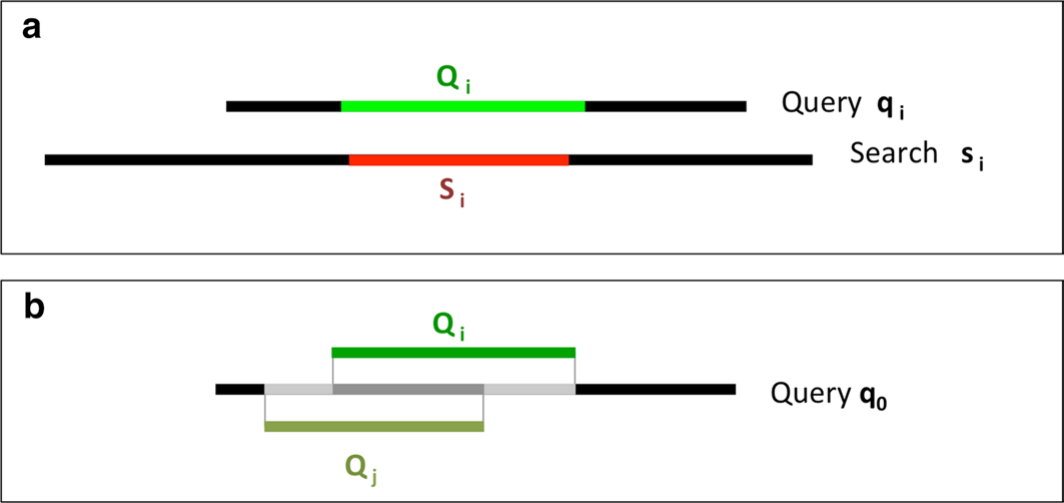
**a** Schematic representation of a pairwise alignment $B_i = ( q_i,s_i,\mathcal {Q}_i,{\mathcal {S}}_i )$. The aligned regions are shown in green (query) and red (search). **b** Representation of two different alignments (*i* and *j*) on the same query $q_0$. The aligned regions on the query are shown in green. The dark-gray portion of the protein represents the intersection between region $\mathcal {Q}_i$ and region $\mathcal {Q}_j$ , namely $\mathcal {Q}_i \cap \mathcal {Q}_j$; ; the dark-gray+light grey region represents union of region $\mathcal {Q}_i$ and region $\mathcal {Q}_j$, namely $\mathcal {Q}_i \cup \mathcal {Q}_j$

We define a *BLAST alignment*, labeled by an index *i*, as:

3 $$\begin{aligned} B_i = \Big ( q_i,s_i,\mathcal {Q}_i,{\mathcal {S}}_i \Big ) \end{aligned}$$

where $q_i$ is the identifier of the query sequence, $s_i$ is the identifier of the search sequence, $\mathcal {Q}_i$ and ${\mathcal {S}}_i$ are regions on, respectively, the query and the search sequence. $\mathcal {Q}_i$ and ${\mathcal {S}}_i$ represent the boundaries (start and end points) of the pairwise alignments on the query and on the search sequences, respectively (see Fig. 10a). Note that gaps and insertions are not taken into account.

### Clustering of BLAST alignments

DPC [22] entails the following steps: (1) defining a distance in the space of the objects that are to be clustered; (2) estimating the local density of each object, namely the probability of observing other objects within a small distance; (3) selecting the objects corresponding to density peaks (cluster centers) and, finally, (4) assigning of non-peak objects to density peaks (clustering). Here we perform two rounds of DPC. The first round allows us clustering alignments that cover similar regions of the query sequences (primary clusters, meant to represent domains); in the second round we group together primary clusters that share a number of overlapping alignments (metaclusters), which are pruned from redundancies in the merging step. Alignments belonging to metaclusters can then be linked back to the respective aligned sequences, thus obtaining clusters of protein regions, which are meant to represent families (see “Results” section).

**Fig. 11:**
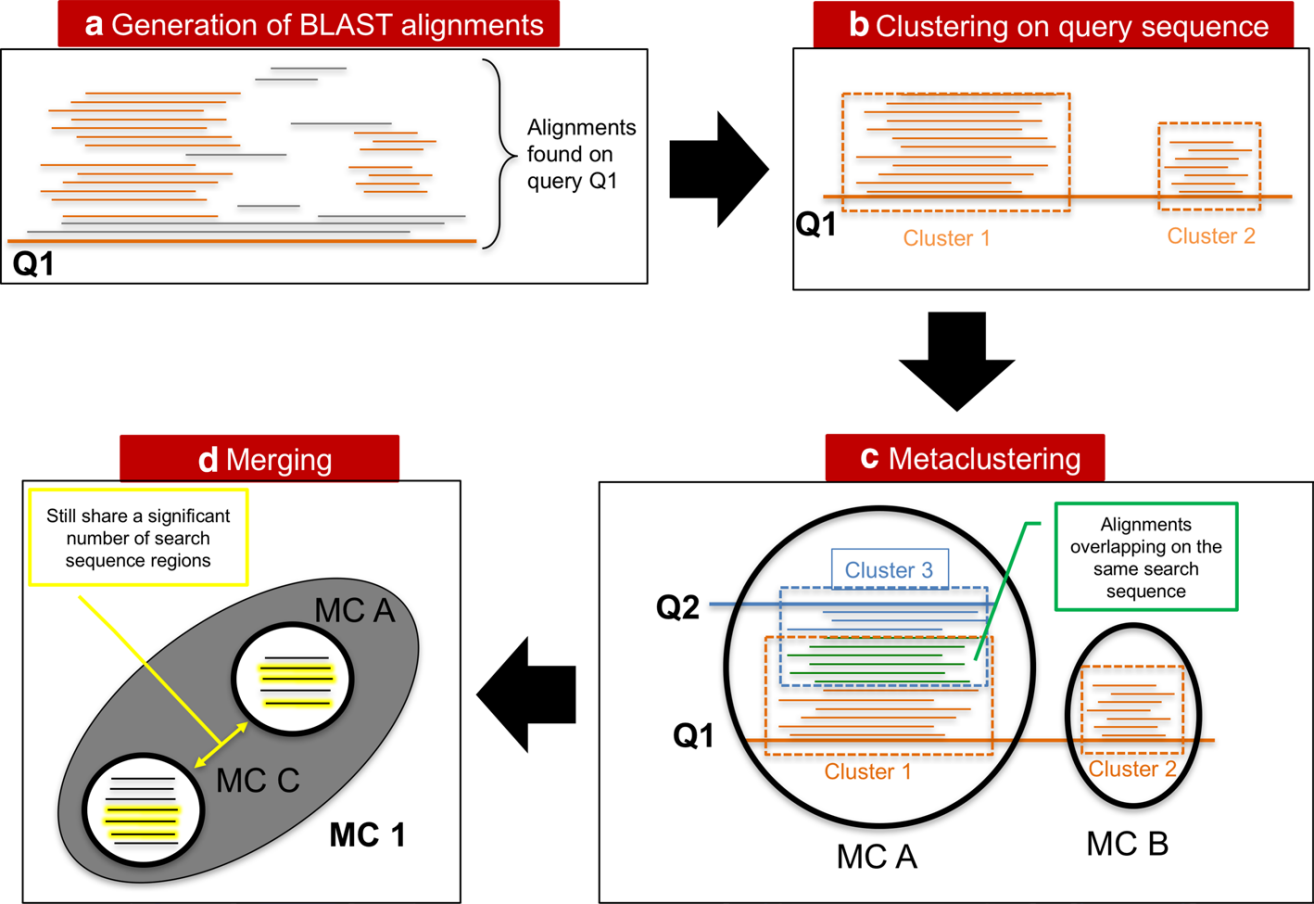
Graphical overview of the method. After running BLAST on a set of query sequences (**a**), alignments that lie on the same region of each query sequence are grouped into primary clusters (**b**); subsequently, primary clusters are “metaclustered” according to the number of search sequence alignments they share (**c**); finally, the set of metaclusters is pruned from redundancies by grouping those that share a significant number of search sequence regions (**d**)

#### Primary clustering

For a query $q_0$ we write the set of all of its alignments as:

4 $$\begin{aligned} \mathcal {B}^{q_0}=\lbrace B_i : q_i=q_0 \rbrace \end{aligned}$$

We define the distance between alignments in $\mathcal {B}^{q_0}$ as:

5 $$\begin{aligned} d_{i,j}^{\mathcal {Q}} = 1- \frac{ |\mathcal {Q}_i \cap \mathcal {Q}_j| }{ | \mathcal {Q}_i \cup \mathcal {Q}_j | } \end{aligned}$$

where $|\mathcal {Q}_i \cap \mathcal {Q}_j|$ is the length (intended as number of residues) of the intersection between the segments identified by $\mathcal {Q}_i$ and $\mathcal {Q}_j$, while $|\mathcal {Q}_i \cup \mathcal {Q}_j|$ is the length of their union (see Fig. 10b). This distance is 0 if $B_i$ and $B_j$ are aligned to the same portion of the query $q_0$, that is, $\mathcal {Q}_i = \mathcal {Q}_j$; while it is 1 if $\mathcal {Q}_i$ and $\mathcal {Q}_j$ do not overlap at all. As defined, $d^{\mathcal {Q}}_{i,j}$ represents a metric since it is symmetric and satisfies the triangular inequality. Using the distance in Eq. 5, we estimate the density $\rho _i$ of the alignment *i*:

6 $$\begin{aligned} \rho _i = \sum _{j} \chi _{\mu _1} ( d^{\mathcal {Q}}_{i,j} ) \end{aligned}$$

where $\chi _{\mu _1} ( x ) =1$ if $x<\mu _1$ and zero otherwise. Thus, the density of an alignment $B_i$ is given by the number of alignments that belong to the same set $\mathcal {B}^{q_0}$ and that are found at a distance less than $\mu _1$ from $B_i$. In the algorithm, we set $\mu _1=0.2$, according to the rule of thumb in [22]: using this threshold the average number of neighbours closer than $\mu _1$ to a point is around 1 to 2% of the total number of points in the dataset. When two alignments with the same search sequence are such that $d^{\mathcal {Q}}_{i,j}<\mu _1$, we retain only the alignment with the lowest E-value (for each query, this happens for 1% or less of the alignments).

Next, following [22] we define $\gamma _i=\delta _i\rho _i$, where $\delta _i=\min _{j: \rho _j>\rho _i}d^{\mathcal {Q}}_{i,j}$ , namely the minimum distance of *i* to a higher density point *j*. Then we sort the alignments according to decreasing values of $\gamma _i$, $\Gamma (q_0)= \left\{ \gamma _s , \;\; \gamma _s> \gamma _{s+1} \;\; \forall s\right\}$. Finally we select density peaks by identifying a $\gamma _g \in \Gamma (q_0)$ such that $\frac{ \gamma _{g-1} }{ \gamma _{g} } \ge 10^{\Delta } \; \; \& \; \frac{ \gamma _{s-1} }{ \gamma _{s} } < 10^{\Delta } \; \forall s>g \; \; \& \; g \le g_{max}$. This is equivalent to looking for a gap of size $\Delta$ between values in $\Gamma (q_0)$ (this was done by eyesight in [22]). We choose heuristically $\Delta =0.5$ and $g_{max}=20$, where $g_{max}$ is the maximum number of peaks (primary clusters, see below) that we allow on a query sequence. The robustness of the results with respect to these parameters is discussed below. As a final step, we assign to each density peak all alignments that are found at a distance smaller than $\mu _1$ from the peak, and further away from any other peak: alignments mapping to a peak constitute what we call a *primary cluster*. Note that, generally, not all $B_i$ alignments are assigned to a primary cluster: we discard the non-clustered alignments in the downstream analysis.

**Fig. 12:**
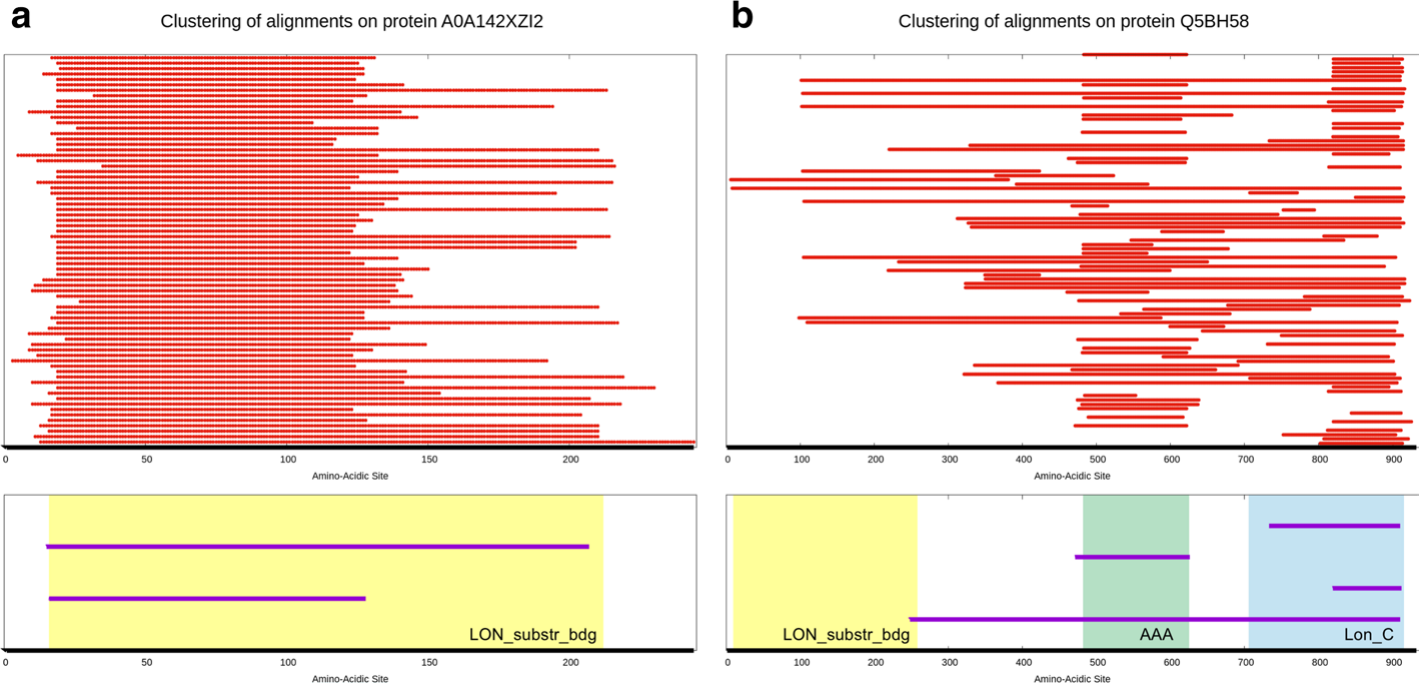
Examples of primary clustering for two proteins in PUA_UR50, namely A0A142XZI2 (**a**) and Q5BH58 (**b**). Thick, black lines represent the query sequences. Red lines show regions of the query that have been aligned by BLAST to other sequences in the PUA_UR50 dataset. The bottom part of each panel shows a comparison between Pfam annotation and MC clustering of the query sequences. According to Pfam, both A0A142XZI2 and Q5BH58, contain a LON_substr_bdg domain (a member of the PUA clan), the position of which is highlighted by a yellow frame. Protein Q5BH58, in addition, contains an AAA domain and a Lon_C domain, colored green and blue, respectively. Purple lines show the primary clusters we obtained automatically using the red line alignments at the top of each panel. Primary clusters are sorted from top to bottom according to decreasing value of their $\gamma$ parameter (see “Methods” section), so that the top ones will most probably be cluster centers. We can see that some of the primary clusters overlap remarkably well with Pfam-annotated families while others either cover more than one family or overlap with only a fraction of a family. Also, note that in Q5BH58 no MC captures the LON_substr_bdg domain. In this particular case, we found that this region of Q5BH58 is a quite divergent member of the Pfam family, with both BLAST and phmmer finding less than 10 parwise alignments when using that portion of the protein as a query

The clusters we obtain are subsets of the previously defined $\mathcal {B}^{q_0}$ set, where each subset includes alignments located around the same region of the query sequence. The clustering procedure we described is schematically shown in Fig. 11a, b, and two examples of primary clustering are shown in Fig. 12.

#### Metaclustering

We denote the set of alignments belonging to a primary cluster *c* as $\mathcal {B}_c$ and we call $N_c$ the number of is elements.

We define the distance between two clusters *c* and $c_0$, associated to two queries *q* and $q_0$ as:

7 $$\begin{aligned} D_{c,c_0}= 1 - \frac{1}{min(N_c,N_{c_0})} \sum _{m \in \mathcal {B}_c, n \in \mathcal {B}_{c_0}} \delta _{s_m s_n} \chi _{\mu _d} ( d^{{\mathcal {S}}}_{m,n} ) \end{aligned}$$

where $d^{{\mathcal {S}}}_{i,j}$ is defined as in Eq. 5 using segments ${\mathcal {S}}_i$ and ${\mathcal {S}}_j$ in place of $\mathcal {Q}_i$ and $\mathcal {Q}_j$, and $\mu _d=0.2$ is chosen coherently with $\mu _1$ in Eq. 6. This distance is small if the number of alignments in the two clusters sharing the same search sequence is high.

We estimate the density $\rho _c$ similarly as in Eq. 6:

8 $$\begin{aligned} \rho _c = \sum _{c'} \chi _{\mu _2}( D_{c,c_0} ) \end{aligned}$$

where $\mu _2=0.9$ was also chosen following the rule of thumb in [22]. Then, similarly to what done in 2.2.1, we compute $\delta _c=\min _{c': \rho _{c'}>\rho _c} D_{c,c'}$. This time, however, we use a more restrictive criterion for the identification of density peaks by choosing as peaks those primary clusters for which $\delta _c$ takes its maximum value of 1, and for which $\rho _c>1$. The reason for this is that different peaks in the primary cluster space should not have significant overlaps between each other. Finally, we assign to each density peak all primary clusters that are found at a distance smaller than $\mu _2$ from the peak, and further away from any other peak; the set of primary clusters assigned to a peak constitute what we call a *metacluster* or *MC*. Primary clusters not assigned to any peak are discarded.

#### Merging metaclusters

The procedure described above produces at times metaclusters which share a significant fraction of sequences, and can therefore be considered redundant. We merge similar MCs by computing the quantity

9 $$\begin{aligned} D_{MC',MC''}= \frac{2}{N_{MC'} N_{MC''} } \sum \limits _{c' \in MC', c'' \in MC''} D_{c',c''} \end{aligned}$$

where $MC'$ and $MC''$ are any two metaclusters and $N_{MC'}$ and $N_{MC''}$ is the number of their primary clusters. $D_{MC',MC''}$ is the average of the distances between primary clusters contained in the two MCs. We merge all MC pairs for which $D_{MC',MC''}<0.9$.

#### Filtering metaclusters’ alignments and building profile-HMMs

A metacluster is a collection of protein regions ${\mathcal {S}}_i$. In order to reduce the level of noise coming from outlier sequences within an MC, from the list of all regions obtained in the previous section we remove those that don’t overlap with any other sequence in the MC. More specifically, we keep region *i* if it exists another region *j* in the same MC such that $\delta _{s_i s_j} \chi _{\mu _d} ( d^{{\mathcal {S}}}_{i,j} ) = 1$ (cfr. Eq. 7). We additionally reduce redundancy at 95 percent identity using CD-HIT [16] (v4.7). MCs that at this stage contain less than 100 elements are removed from downstream analysis, since our approach can only identify clusters the population of which is large enough to form a density peak, which can be reasonably distinguished from the background noise. In our comparison with Pfam annotation, only up to 5000 members per MC are taken into consideration; if an MC has > 5000 members, we select 5000 randomly to represent it. For the purpose of building MC-associated profile-HMMs, we further reduce MCs’ size by reducing redundancy at 60% (using CD-HIT [16]) and considering maximum 1000 members (if > 1000, we select 1000 randomly). Next, we build an MSA using MUSCLE [34] and use the MSA to construct a profile-HMM, using HMMER (v3.1b) [24]. We note that HMMER trims poorly ’populated’ N- and C-terminal regions of MSAs by considering as match states of the model only columns containing $\ge$ 50% sequences (see documentation at http://eddylab.org/software/hmmer3/3.1b2/Userguide.pdf). Although our choice of an E-value threshold equal 0.1 is expected to produce a certain number of false positives, the tradeoff with sensitivity means that we also gather a larger number of true positive relations. We expect clustering not to be affected by a small number of unrelated false positives (note that we perform single BLAST runs thus preventing false positive propagation at the alignment stage); however, the systematic misalignment of two unrelated families might lead to generation of erroneous clusters. Although in the experiments performed in this study we have not noticed any such occurrence, we cannot exclude that they will be observed when processing larger, more diverse sequence datasets.

### Robustness of the metaclustering procedure

We test (*a posteriori*) the robustness of the metaclustering procedure on P53_UR50 with respect to small variations ($\pm 10\%$) of the $\mu _1$, $\mu _2$ and $\Delta$ parameters, and when reducing by half the size of the query sequence dataset. In particular, we compare the assignment of alignments to metaclusters before the filtering step (see Additional file 1: Table S1).

In our comparison, we use: (1) the number of alignments that are assigned to metaclusters; (2) the percentage of alignments metaclustered with the standard set of parameters that are still assigned to metaclusters when utilising the modified parameters; (3) the Normalized Mutual Information. The NMI is given by $\text {NMI}(C_1,C_2)= \frac{2 I(C_1,C_2)}{H(C_1)+H(C_2))} \in [0,1]$, where $C_1$ and $C_2$ are the class labels assigned to alignments by two different clustering procedures, *I* is the Mutual Information between the two classifications and *H*(*C*) is the entropy of a single classification. Two identical classifications gives NMI$=1$. To compute NMI, we consider those alignments that have been metaclustered by both the reference and the alternative clustering procedure (i.e, those counted in the third column of Additional file 1: Table S1).

In general, parameters’ variation does not result in significant changes in the number of alignments assigned to a metacluster. Variations in $\mu _1$ and $\mu _2$ imply smaller or larger cutoffs in the density estimations, and a more or less restrictive criterion for assigning alignments to cluster centers. Not surprisingly, larger values of $\mu _1$ and $\mu _2$ produce larger metacluetsers, while smaller values produce smaller metaclusters (see second column of Additional file 1: Table S1). This is reflected also in the percentage of alignments which are metaclustered using the standard procedure that are also retrieved when varying $\mu _1$ and $\mu _2$, with smaller percentages obtained using smaller values (see third column of Additional file 1: Table S1). However, despite of these differences, the NMI with the results obtained with the reference setup is always extremely high, indicating that the results are robust with respect to the choice of this parameter.

Different values of $\Delta$ result in adding or removing density peaks: the small variations performed do not change significantly the number of alignments metaclustered (changes of about 2%), covering the vast majority (98%) of the alignments clustered with the standard procedure. Also in this case the NMI with respect to the reference setup is very high.

We also repeated the whole procedure on a query dataset containing only half of the sequences, selected at random (50% P53_UR50). In this analysis we collect 642,223 alignments in metaclusters. In the same subset of sequences, performing the analysis on the full dataset we assign to metaclusters 644,648 alignments. Almost all these alignments are in common (see third column of Additional file 1: Table S1) and, consistently, the NMI between the two metacluster partitions is 0.99.

## Supplementary Information

**Additional file 1.** Supplementary Information cited through the manuscript.

## Data Availability

The datasets supporting the conclusions of this article are available in the Zenodo repository, https://zenodo.org/record/4114672 [35]. The software used to generate metaclusters is available at https://gitlab.com/ETRu/dpcfam (Requires Python 3, C++ compiler and runs on Linux systems; GNU GPL).

## Abbreviations

DPC: Density peak clustering
MSA: Multiple sequence alignment
HMM: Hidden Markov model
MC: MetaCluster
GTA: Ground truth architecture
DA: Dominant architecture
%DAF: Percentage of dominant architecture (family level)
%DAC: Percentage of dominant architecture (clan level)
%DACF: Percentage of dominant architecture (clan level) and fewer
%DACFA: Percentage of dominant architecture (clan level), fewer and additional
