# Supplementary material for: Density Peak clustering of protein sequences associated to a Pfam clan reveals clear similarities and interesting differences with respect to manual family annotation
Source: BMC Bioinformatics. 2021 Mar 12;22:121. doi: 10.1186/s12859-021-04013-x (PMC7955657; doi:10.1186/s12859-021-04013-x)
Supplement: Supplementary file 1 — Additional file 1. Supplementary Information cited through the manuscript. [file 12859_2021_4013_MOESM1_ESM.pdf]

Density Peak clustering of protein sequences associated to a Pfam clan reveals clear similarities and interesting differences with respect to manual family annotation - SUPPLEMENTARY INFORMATION

E.T. Russo, A. Laio., M.Punta

October 21, 2020

1 Supplementary Tables

**Parameters**	**Number of alignments metaclustered**	**Percentage of $\mu_1 = 0.2$, $\mu_2 = 0.9$, $\Delta = 0.5$ alignments' metaclustered**	**NMI over common alignments**
$\mu_1 = 0.2$, $\mu_2 = 0.9$, $\Delta = 0.5$	1,350,496	-	-
$\mu_1 = 0.2 + 10\%$	1,484,231	94%	0.96
$\mu_1 = 0.2 - 10\%$	1,351,613	89%	0.99
$\mu_2 = 0.9 + 10\%$	1,494,990	95%	0.99
$\mu_2 = 0.9 - 10\%$	1,259,045	93%	0.99
$\Delta = 0.5 + 10\%$	1,326,511	98%	0.99
$\Delta = 0.5 - 10\%$	1,374,507	98%	0.95
**Query Dataset**	**Number of 50% P53_UR50 alignments metaclustered**	**Percentage of 644,648 alignments metaclustered**	**NMI over common alignments**
P53_UR50	644,648	-	-
50%_P53_UR50	642,223	99%	0.99

Table S1: *Metaclusters' robustness upon ±10% variation of the μ_1, μ_2, Δ parameters and, additionally, when reducing by half the number of query sequences.* Test are performed on the P53_UR50 dataset. We consider alignments assigned to metaclusters before the filtering step (see Methods). $\mu_1 = 0.2$, $\mu_2 = 0.9$, $\Delta = 0.5$ are the parameters used throughout the manuscript. NMI stands for Normalized Mutual Information (see Results for the definition).

2 Supplementary Figures

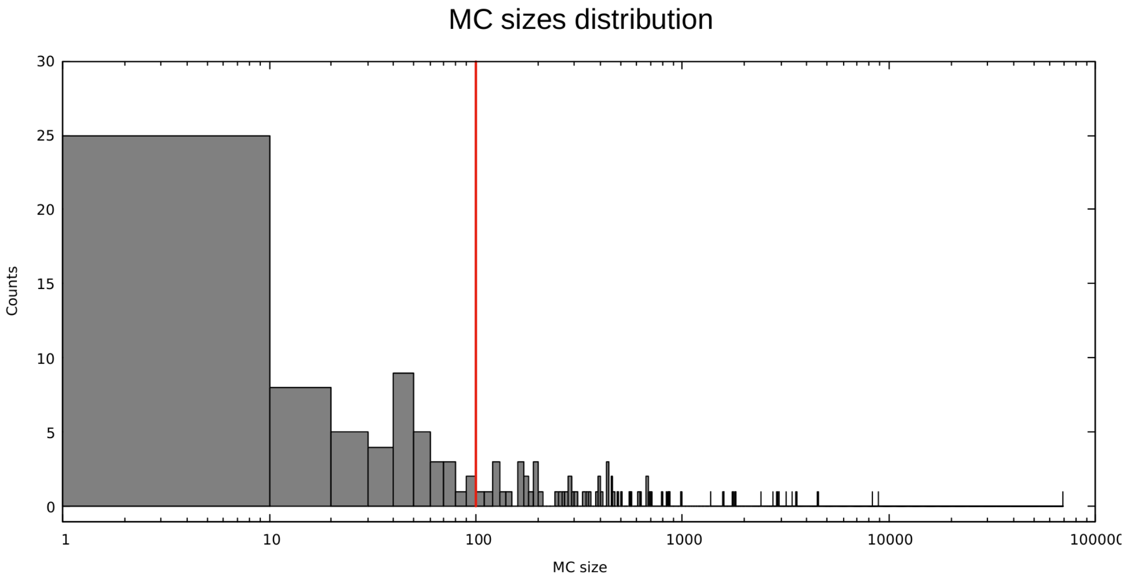

Figure S1: Size distribution of PUA_UR50 metaclusters after redundancy reduction at 95% sequence identity. Here, we include also MCs with less than 100 elements.

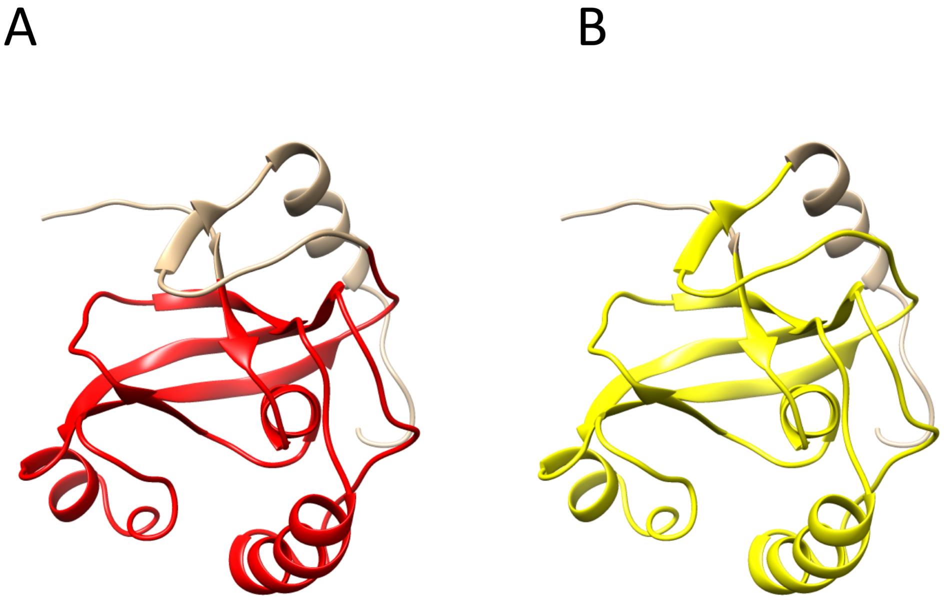

Figure S2: Structure of PDB protein chain 2e5o_A(NMR model #0.1). (A) We highlight in red the Pfam ASCH annotation, roughly corresponding to the boundaries of the "ASC-1 proper family" as found in Iyer *et al.*. It can be seen that the final strand-helix motif is missing from the annotation. (B) We highlight in yellow the region captured by MC-4_PUA profile-HMM, which captures the whole ASCH region, plus the extra strand. Annotation according to Pfam v32.0.

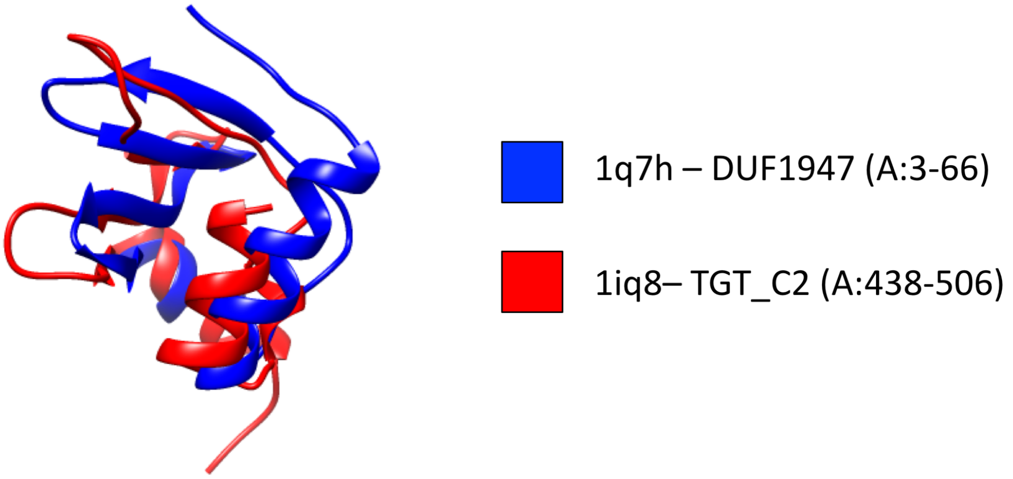

Figure S3: Structural alignment of DUF1947 domain is pdb structure 1q7h (A:3-66) (protein Q9HIB8) with TGT_C2 domain in pdb 1iq8 (A:438-506) (protein O58843). Aligned with Dali (Holm, 2019) ; Z 4.5 , rmsd 3.0, nres 60 and %ID 12 .

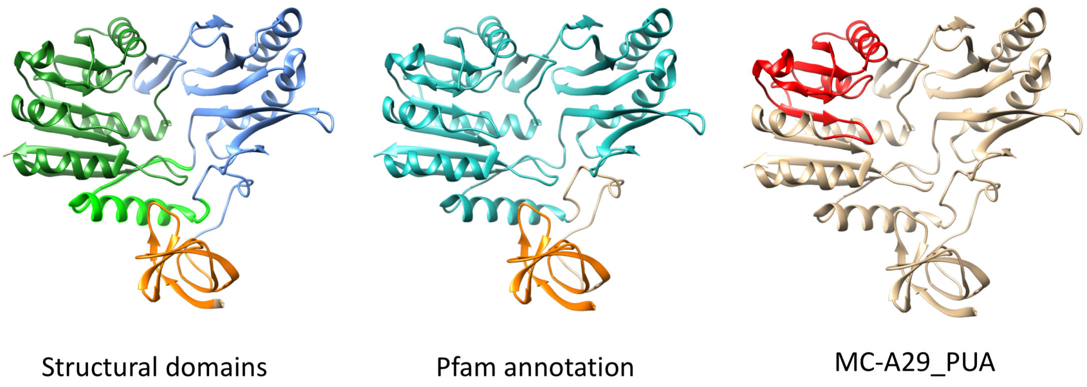

Figure S4: X-ray crystal structure of RumA, an E.coli class I SAM-dependent methyltransferase (PDB:2bh2_A). Structural domains (following (Lee et al., 2004): N-terminal domain (orange, aa15-74), Central domain (light blue, aa75–92 and 125–262) and C-terminal (catalytic) domain (light green, aa93–124 and green, aa263–431); (center) Pfam annotation: TRAM (PF01938) (cyan, aa10-67) and tRNA_U5-meth_tr (PF05958) (orange, aa95-432); (right) Region that aligns (HMMER online v3.3) to the profile-HMM built of MC-A29_PUA (aa285-369, red).

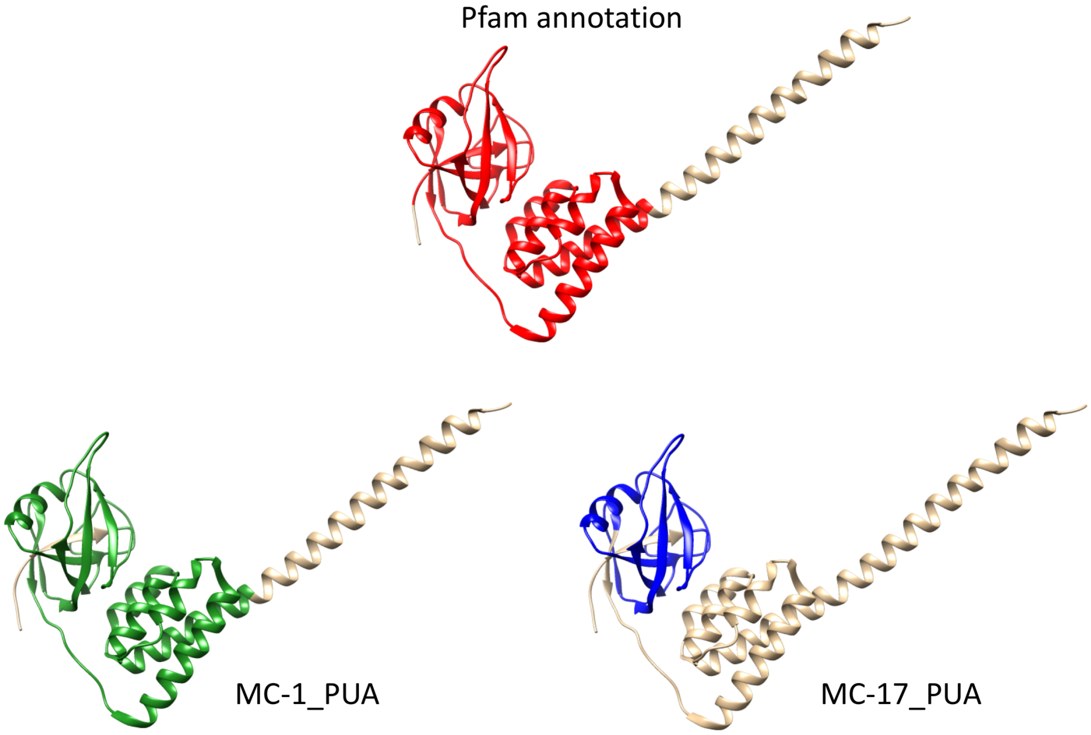

Figure S5: Structure of PDB protein chain 3ljc_A (protein P0A9M0). Red section show Pfam annotation (Lon_substr_bdg aa. 10-202). Green region (aa. 17-207) shows the hit of MC-1_PUA profile-HMM (E-value 2.2E-26); blue region (aa. 17-109) shows the hit of MC-17_PUA profile-HMM (E-value 1.1E-5).

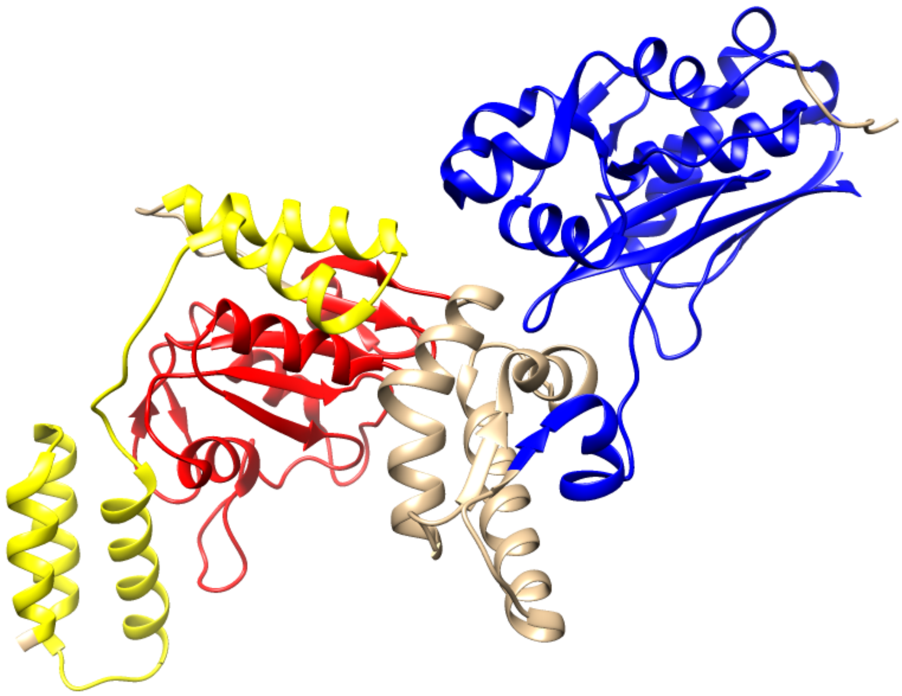

Figure S6: Structure of PDB protein chain 4ypl_A (protein A0A059VAZ3). Red and blue section show Pfam annotation: AAA region in red (aa. 351-491) and LON_C in blue (aa. 568-772). Yellow region (aa. 245-339) shows the hit of MC-A48_PUA profile-HMM.

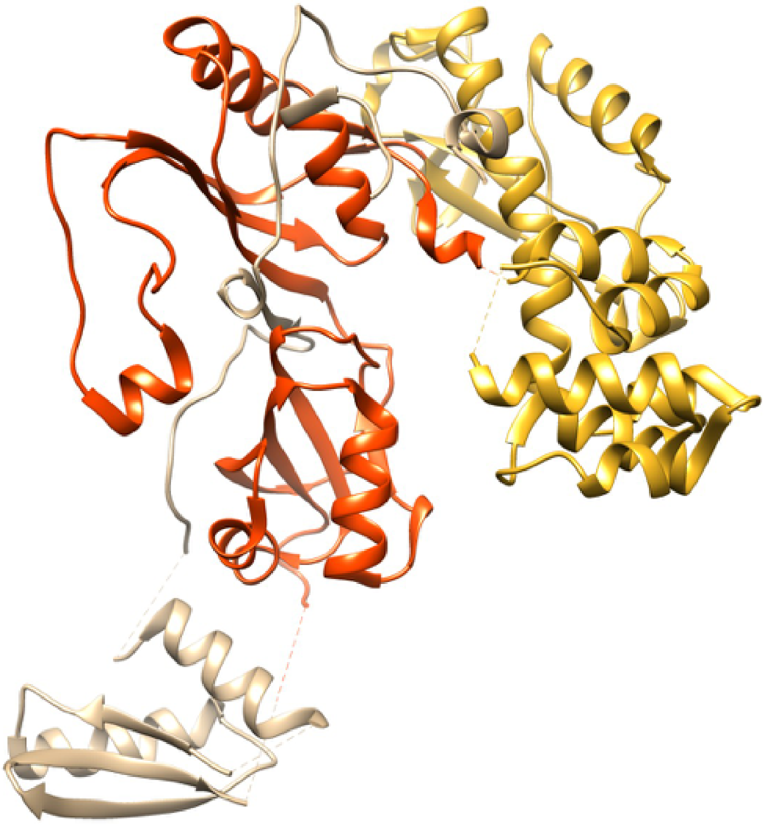

Figure S7: Structure of PDB protein chain 5kkp_A. The colored region (red+gold) covers a TruD domain as annotated in Pfam. The nested gold domain (roughly, aa384-577) is not related in structure to domains in the PseudoU_synth clan and, as such, should be built as a separate Pfam family not part of the clan. Regions not annotated in Pfam are colored tan. TruD nnotation according to Pfam 32.0.

.

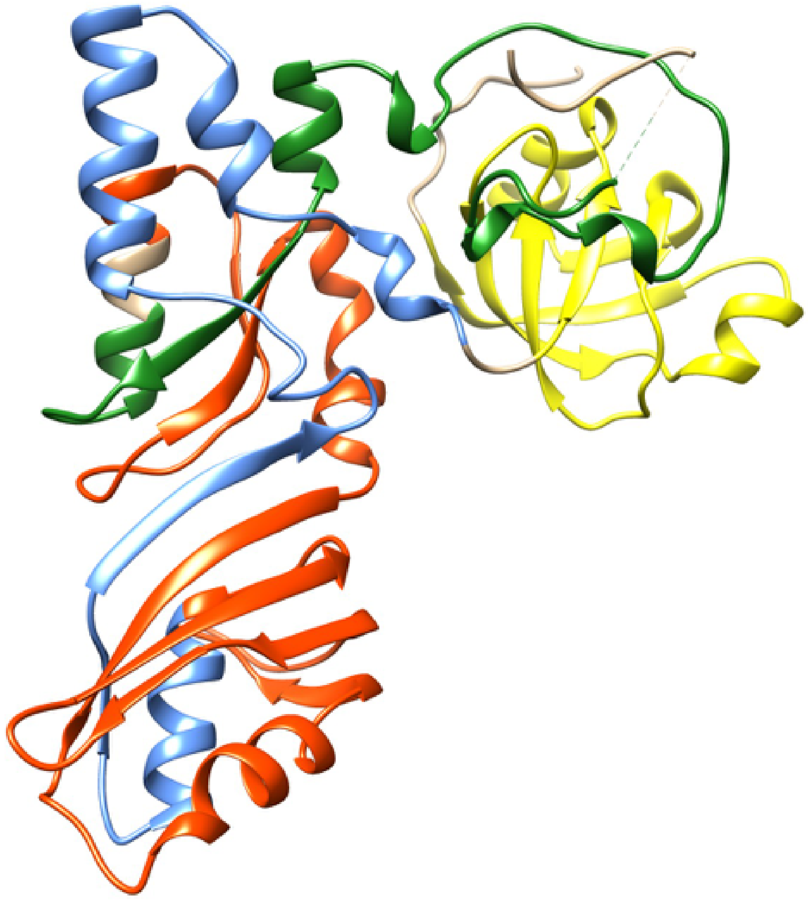

Figure S8: Structure of PDB protein chain 3u28_A. Colors identify Pfam families annotated on the structure (from N- to C-terminus): DKCLD (green), TruB_N (red), TruB_C_2 (blue) and PUA (yellow). Regions not annotated in Pfam are colored tan. Annotation according to Pfam 32.0.

.

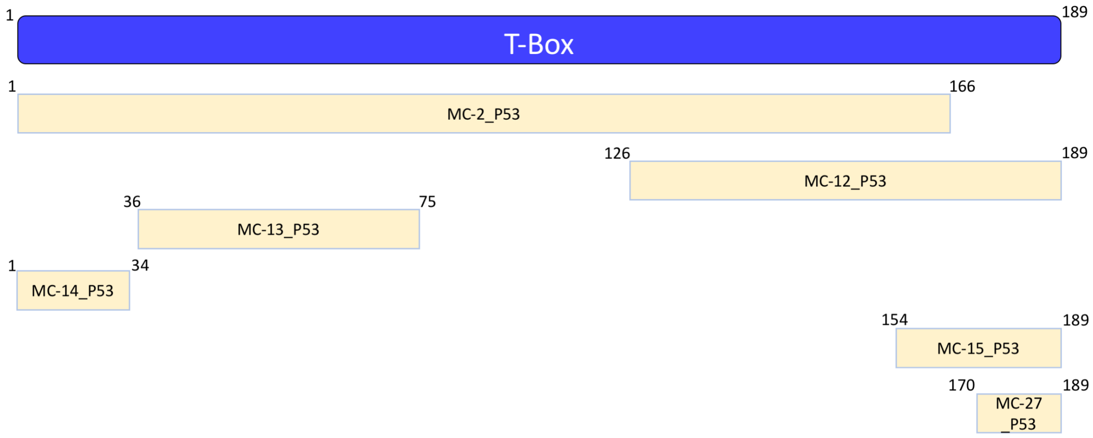

Figure S9: Coverage of P53_UR50 redundant metaclusters with respect to their common PF00907 (T-Box) DA. We used HHpred to determine the position of each MC with respect to the T-box profile-HMM (the first match for all these MCs, with hhpred probability>98%).

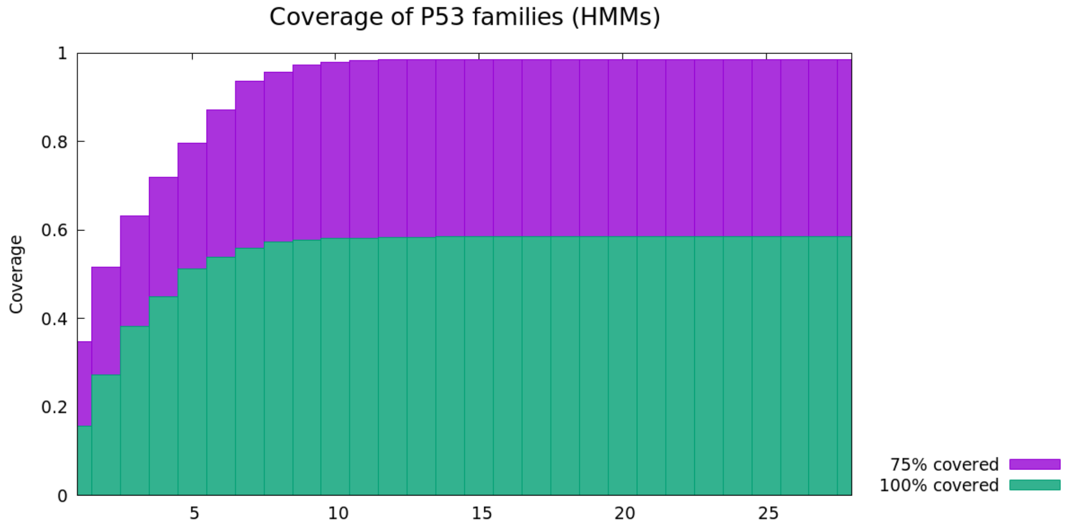

Figure S10: Coverage of proteins in UniRef50 including at least a region of the P53-like clan according to Pfam-A. By running the profile-HMMs derived from the metaclusters, we search for hits with at least 75% of coverage and 100% of coverage. The graph shows the fraction of the P53 clan covered using an increasing number of metaclusters.
